# Burdens of post-acute sequelae of COVID-19 by severity of acute infection, demographics and health status

**DOI:** 10.1038/s41467-021-26513-3

**Published:** 2021-11-12

**Authors:** Yan Xie, Benjamin Bowe, Ziyad Al-Aly

**Affiliations:** 1 Clinical Epidemiology Center, Research and Development Service, VA Saint Louis Health Care System, Saint Louis, MO USA; 2 Veterans Research and Education Foundation of Saint Louis, Saint Louis, MO USA; 3 Department of Epidemiology and Biostatistics, College for Public Health and Social Justice, Saint Louis University, Saint Louis, MO USA; 4 Department of Medicine, Washington University School of Medicine, Saint Louis, MO USA; 5 Institute for Public Health, Washington University in Saint Louis, Saint Louis, MO USA; 6 Nephrology Section, Medicine Service, VA Saint Louis Health Care System, Saint Louis, MO USA

**Keywords:** Viral infection, SARS-CoV-2, Epidemiology, Outcomes research

## Abstract

The Post-Acute Sequelae of SARS-CoV-2 infection (PASC) have been characterized; however, the burden of PASC remains unknown. Here we used the healthcare databases of the US Department of Veterans Affairs to build a cohort of 181,384 people with COVID-19 and 4,397,509 non-infected controls and estimated that burden of PASC—defined as the presence of at least one sequela in excess of non-infected controls—was 73.43 (72.10, 74.72) per 1000 persons at 6 months. Burdens of individual sequelae varied by demographic groups (age, race, and sex) but were consistently higher in people with poorer baseline health and in those with more severe acute infection. In sum, the burden of PASC is substantial; PASC is non-monolithic with sequelae that are differentially expressed in various population groups. Collectively, our results may be useful in informing health systems capacity planning and care strategies of people with PASC.

## Abstract (web summary)

Post-acute sequelae of COVID-19 (PASC, or ‘Long COVID’) has caused concern but the burden and risk factors are not well understood. Here, the authors use US electronic health record data and estimate an overall burden of PASC of ~7% at six months, with variation by severity of acute infection, baseline health status and demographics.

## Introduction

Emerging reports suggest that beyond the acute illness, some COVID-19 survivors experience myriad clinical abnormalities lasting well beyond the first 30 days of infection^1–7^. We recently developed a high-dimensional approach to comprehensively and systematically characterize the post-acute sequelae of SARS-CoV-2 infection (PASC)—also referred to as post-COVID-19 syndrome, or simply long COVID^1^. Our approach identified 33 sequelae in the pulmonary and several extrapulmonary organ systems including nervous system and neurocognitive disorders, mental health disorders, metabolic disorders, cardiovascular disorders, gastrointestinal disorders, and several other clinical manifestations^1^.

However, significant knowledge gaps remain. Specifically, what is the burden of PASC—defined as having at least one post-acute sequela? And does the burden of individual sequelae differ by age, race, sex, and baseline health status? Addressing these questions has been highlighted as an urgent research priority by stakeholders including the World Health Organization, the United States National Institute of Health, the United Kingdom National Institute for Health and Care Excellence, and several others^2,8–13^. Addressing these questions will help inform capacity planning and care of people with PASC.

Here, we answer this call for urgent research, where we leverage the breadth and depth of the US Department of Veterans Affairs (VA) electronic healthcare databases, which operates the largest integrated health care delivery system in the US, to undertake comprehensive large-scale analyses of 181,384 people with COVID-19 who survived the first 30 days of infection and 4,397,509 non-infected controls, and aimed to estimate the burden of PASC in the overall cohort and among non-hospitalized (*n* = 155,987), hospitalized (*n* = 19,359), and those admitted to intensive care (*n* = 6038) and burdens of the 33 individual sequelae that comprise PASC, identified in prior work^1^, in population groups (by age, race, sex, and baseline health status).

## Results

We enrolled 181,384 veterans who survived the first 30 days of COVID-19 including 155,987, 19,359, and 6038 who were non-hospitalized, hospitalized, and admitted to intensive care during the acute phase (the first 30 days) of the COVID-19 infection. The control group included 4,397,509 users of the Veterans Health Administration (VHA) with no known COVID-19 infection. The baseline demographic and health characteristics are described in Table 1. The unadjusted numbers and percentages of incident clinical manifestations in the control group (VHA users), in the overall COVID-19 cohort, and according to COVID-19 care setting (non-hospitalized, hospitalized, and admitted to intensive care) during the follow-up are presented in Supplementary Table 1.

**Table 1:** Characteristics of (1) users of Veterans Health Administration (VHA), (2) non-hospitalized people with COVID-19, (3) hospitalized COVID-19 but not admitted to intensive care, and (4) hospitalized COVID-19 admitted to intensive care.

	VHA users *n* = 4,397,509	COVID-19 without hospitalization *n* = 155,987	Hospitalized COVID-19 without admit to intensive care *n* = 19,359	COVID-19 admitted to intensive care *n* = 6038
Age (IQR)	67.13	62.21	70.46	70.79
	(53.12, 74.48)	(49.63, 72.60)	(61.03, 76.05)	(62.26, 75.66)
Race, White (%)	3,369,525 (76.62)	112,637 (72.21)	12,320 (63.64)	3755 (62.19)
Race, Black (%)	821,787 (18.69)	35,945 (23.04)	5972 (30.85)	1930 (31.96)
Race, other (%)	206,197 (4.69)	7405 (4.75)	1067 (5.51)	353 (5.85)
Sex, male (%)	3,978,601 (90.47)	137,767 (88.32)	18,197 (94.00)	5714 (94.63)
Sex, female (%)	418,908 (9.53)	18,220 (11.68)	1162 (6.00)	324 (5.37)
Long-term care (%)	26,246 (0.60)	4090 (2.62)	1900 (9.81)	537 (8.89)
Number of outpatient encounter (IQR)^a^	2 (1, 3)	2 (2, 3)	6 (4, 8)	6 (4, 8)
Number of hospital admission (IQR)^a^	0 (0, 0)	0 (0, 0)	0 (0, 1)	0 (0, 1)
Number of prescriptions received (IQR)^a^	5 (3, 11)	8 (4, 14)	13 (7, 20)	14 (8, 20)
Number of outpatient eGFR measurements (IQR)^a^	1 (0, 2)	1 (1, 2)	3 (1, 6)	3 (1, 7)
Area Deprivation Index (IQR)	53.91	54.31	53.92	54.75
	(41.89, 62.66)	(43.52, 62.97)	(42.87, 61.31)	(42.87, 62.20)
Charlson Comorbidity Index (IQR)	0 (0, 1)	1 (0, 2)	2 (1, 4)	2 (1, 5)
Anxiety (%)	376,584 (8.56)	17,523 (11.23)	3711 (19.17)	1113 (18.43)
Cancer (%)	281,992 (6.41)	11,164 (7.16)	2778 (14.35)	885 (14.66)
Cardiovascular disease (%)	577,784 (13.14)	23,145 (14.84)	5779 (29.85)	2025 (33.54)
Chronic kidney disease (%)	594,808 (13.53)	24,522 (15.72)	6344 (32.77)	2140 (35.44)
Chronic lung disease (%)	495,853 (11.28)	20,816 (13.34)	4609 (23.81)	1638 (27.13)
Cerebrovascular disease (%)	197,540 (4.49)	8556 (5.49)	2606 (13.46)	762 (12.62)
Dementia (%)	138,709 (3.15)	7162 (4.59)	2619 (13.53)	700 (11.59)
Depression (%)	305,704 (6.95)	15,671 (10.05)	2919 (15.08)	831 (13.76)
Diabetes mellitus (%)	1,040,084 (23.65)	46,737 (29.96)	8868 (45.81)	3018 (49.98)
Hepatitis C (%)	12,833 (0.29)	599 (0.38)	233 (1.20)	68 (1.13)
Hyperlipidemia (%)	1,157,588 (26.32)	53,080 (34.03)	6435 (33.24)	2108 (34.91)
Hypertension (%)	1,183,516 (26.91)	49,145 (31.51)	7830 (40.45)	2337 (38.70)
Peripheral artery disease (%)	40,250 (0.92)	1800 (1.15)	664 (3.43)	219 (3.63)
Overweight (%)	1,544,583 (35.12)	50,012 (32.06)	5872 (30.33)	1763 (29.20)
Obesity (%)	2,019,446 (45.92)	85,578 (54.86)	9408 (48.60)	3148 (52.14)
Never smoke (%)	2,211,177 (50.28)	84,853 (54.40)	9576 (49.47)	2979 (49.34)
Former smoker (%)	1,151,586 (26.19)	43,231 (27.71)	6020 (31.10)	1900 (31.47)
Current smoker (%)	1,034,746 (23.53)	27,903 (17.89)	3763 (19.44)	1159 (19.20)
Follow-up days (IQR)	150 (115, 221)	149 (114, 209)	155 (117, 262)	173 (124, 282)
Total person-years (sum)	2,112,246.94	73,438.22	9940.89	3336.82

^a^Data collected within 1 year before the cohort enrollment.

**Fig. 1:**
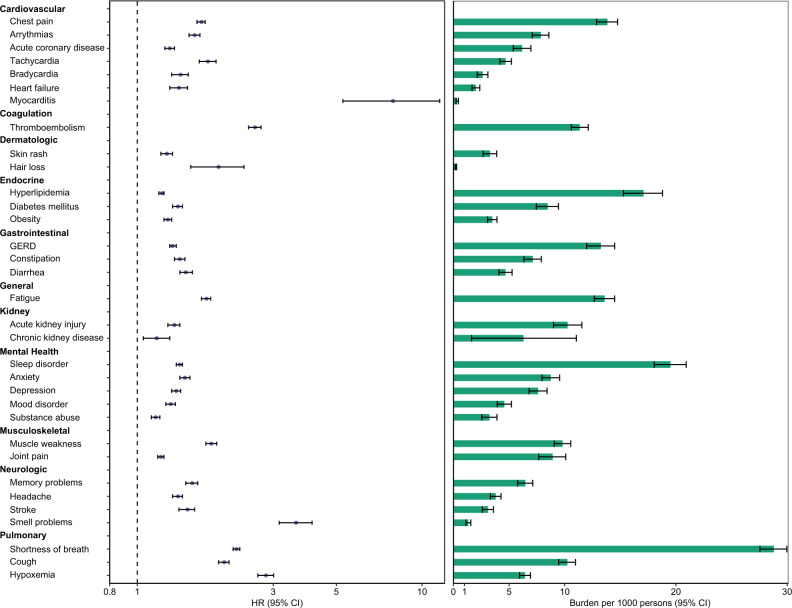
Risks and burdens of post-acute sequelae of SARS-CoV-2 infection (PASC). Organ systems are presented in bolded font. Post-acute sequelae were ascertained from 30 days after infection until the end of follow-up. Hazard ratio and 95% confidence intervals are presented in the left panel and burdens per 1000 COVID-19 patients at 6 months are presented in the right panel; right limit of the bar represents estimated burden and error bars represent the 95% confidence interval. Models were adjusted for age, race, sex, receipt of long-term care, Area Deprivation Index, number of outpatient encounters, number of hospital admissions, number of outpatient prescriptions, number of outpatient serum creatinine measurements, chronic lung disease, cancer, cardiovascular disease, cerebrovascular disease, dementia, diabetes mellitus, hypertension, hyperlipidemia, depression, anxiety, chronic kidney disease, hepatitis C and peripheral artery disease, overweight, obesity, smoking status, Charlson Comorbidity Index, US geographic region, total number of beds, number of COVID-19 tests administered, COVID-19 positivity rate, and average hospital bed occupancy during the week of participant enrollment.

In adjusted analyses, 30-day survivors of COVID-19 exhibited increased risk of a broad range of incident sequelae including pulmonary system disorders and cardiovascular, coagulation, dermatologic, endocrine, gastrointestinal, kidney, mental health, musculoskeletal, and neurologic system disorders; risks and associated burdens for each sequela are provided in Fig. 1 and Supplementary Table 2.

### Burden of PASC according to care setting of the acute infection

**Fig. 2:**
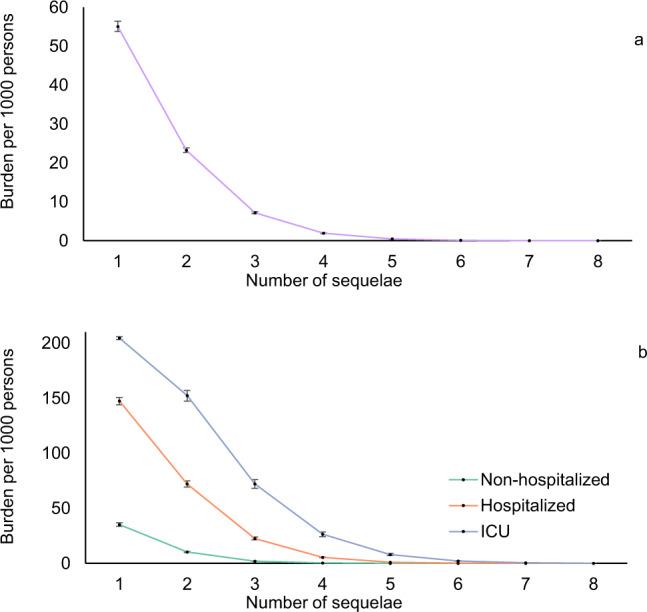
Burden of post-acute sequelae of COVID-19 as a function of the number of sequelae. **a** Overall cohort (purple), and **b** by care setting (non-hospitalized (green), hospitalized (orange), and admitted to intensive care (blue) during the acute phase of the infection). Post-acute sequelae were ascertained from 30 days after infection until the end of follow-up. Estimates of burdens per 1000 COVID-19 patients at 6 months are presented; line represent the estimated burden and error bars represent the 95% confidence interval for the corresponding number of sequelae. Models were adjusted for age, race, sex, receipt of long-term care, Area Deprivation Index, number of outpatient encounters, number of hospital admissions, number of outpatient prescriptions, number of outpatient serum creatinine measurements, chronic lung disease, cancer, cardiovascular disease, cerebrovascular disease, dementia, diabetes mellitus, hypertension, hyperlipidemia, depression, anxiety, chronic kidney disease, hepatitis C and peripheral artery disease, overweight, obesity, smoking status, Charlson Comorbidity Index, US geographic region, total number of beds, number of COVID-19 tests administered, COVID-19 positivity rate, and average hospital bed occupancy during the week of participant enrollment.

To gain a better understanding of the toll of long covid in 30-day survivors of acute COVID-19, we first estimated the burden of PASC—defined as having at least one sequela in excess of the control group (VHA users without COVID-19). Unadjusted and adjusted burdens are presented in Supplementary Table 3 and Table 2, respectively. The adjusted burden of PASC in the overall cohort was 73.43 (72.10, 74.72) per 1000 persons at 6 months (Table 2), and increased according to care setting of the acute infection and was 44.51 (43.09, 45.85), 217.08 (212.43, 222.23), and 360.16 (350.53, 369.38) per 1000 persons at 6 months among non-hospitalized, hospitalized, and those who were admitted to intensive care during the first 30 days of infection, respectively (Table 2 and Fig. 2).

**Table 2:** Overall burden of post-acute sequelae of SARS-CoV-2 infection (PASC) per 1000 persons at 6 months in the overall cohort and across care setting (non-hospitalized, hospitalized, and admitted to intensive care during the acute phase of the infection).

	COVID-19 group	Users of the VHA without COVID-19	Burden associated with COVID-19^b^
	Adjusted burden per 1000 persons at 6 months (95% CI)	Adjusted burden per 1000 persons at 6 months (95% CI)	Adjusted burden per 1000 persons at 6 months (95% CI)
Overall	168.01	94.56	73.43
	(166.68, 169.29)	(94.23, 94.88)	(72.10, 74.72)
Non-hospitalized COVID-19^a^	139.07	94.56	44.51
	(137.63, 140.44)	(94.23, 94.88)	(43.09, 45.85)
Hospitalized COVID-19^a^	311.61	94.56	217.08
	(306.90, 316.82)	(94.23, 94.88)	(212.43, 222.23)
COVID-19 admitted to ICU^a^	454.74	94.56	360.16
	(445.01, 463.92)	(94.23, 94.88)	(350.53, 369.38)

^a^Care settings during the first 30 days of infection.

^b^Burden defined as having at least one sequela in excess of users of the Veterans Health Administration without COVID-19 after the first 30 days of infection. Burdens were estimated based on Poisson regressions adjusting for age, race, sex, receipt of long-term care, Area Deprivation Index, number of outpatient encounters, number of hospital admissions, number of outpatient prescriptions, number of outpatient serum creatinine measurements, chronic lung disease, cancer, cardiovascular disease, cerebrovascular disease, dementia, diabetes mellitus, hypertension, hyperlipidemia, depression, anxiety, chronic kidney disease, hepatitis C and peripheral artery disease, overweight, obesity, smoking status, Charlson Comorbidity Index, US geographic region, total number of beds, number of COVID-19 tests administered, COVID-19 positivity rate, and average hospital bed occupancy during the week of participant enrollment.

### Burden of individual sequelae by age, race, sex, and health status

**Fig. 3:**
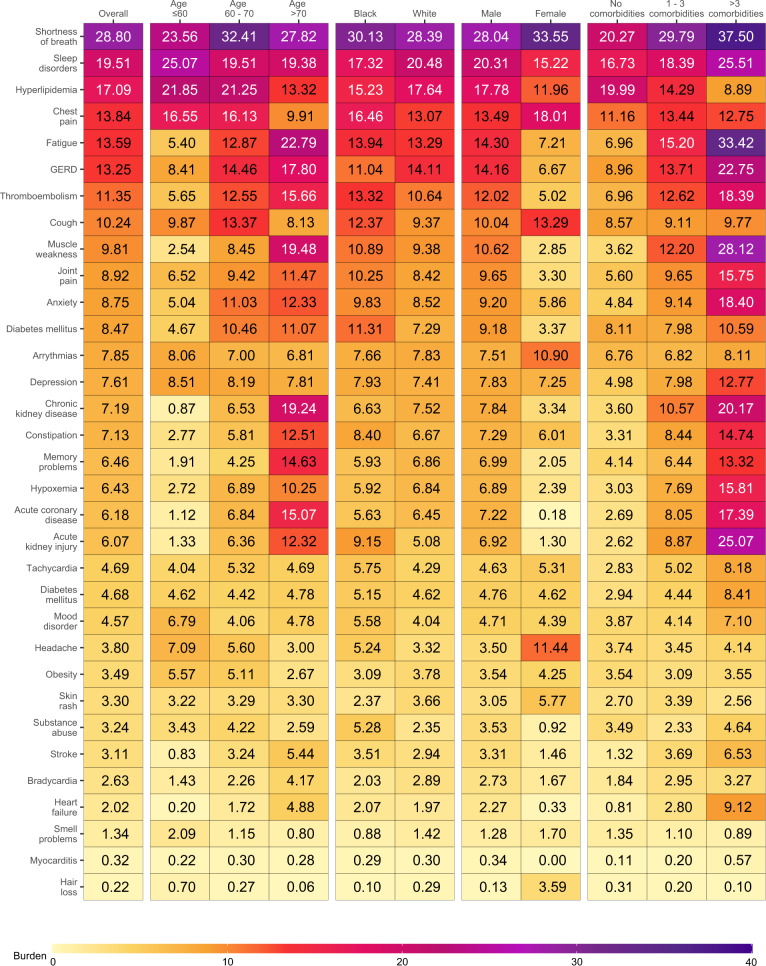
Burden of post-acute sequelae of COVID-19 in the overall cohort and by age, race, sex, and health status. Post-acute sequelae were ascertained from 30 days after infection until the end of follow-up. Estimates of burdens per 1000 COVID-19 patients at 6 months are presented. Models were adjusted for age, race, sex, receipt of long-term care, Area Deprivation Index, number of outpatient encounters, number of hospital admissions, number of outpatient prescriptions, number of outpatient serum creatinine measurements, chronic lung disease, cancer, cardiovascular disease, cerebrovascular disease, dementia, diabetes mellitus, hypertension, hyperlipidemia, depression, anxiety, chronic kidney disease, hepatitis C and peripheral artery disease, overweight, obesity, smoking status, Charlson Comorbidity Index, US geographic region, total number of beds, number of COVID-19 tests administered, COVID-19 positivity rate, and average hospital bed occupancy during the week of participant enrollment when appropriated.

**Fig. 4:**
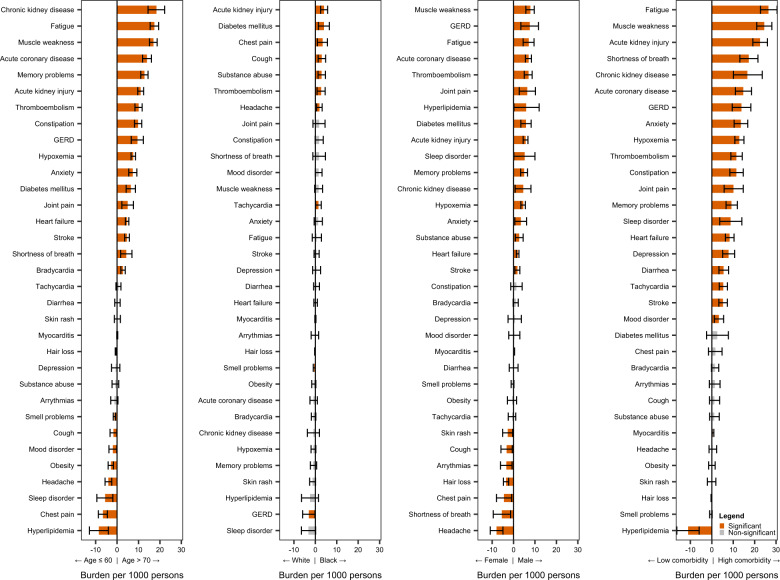
Differences in burden of individual post-acute sequelae of COVID-19 by age, race, sex, and health status. Differences in burden per 1000 COVID-19 patients at 6 months are presented along with 95% confidence intervals.

To gain a better understanding of whether and to what extent the burden of each sequelae may be differentially expressed across various population groups, we estimated the burden of incident individual post-acute sequelae per 1000 persons at 6 months (the burden in excess of the control group) by age, race, sex, and baseline health status represented by the number of comorbidities at baseline (Fig. 3 and Supplementary Tables 4 and 5). This analysis showed that the burdens were not uniformly expressed across age, race, sex, and baseline health status. Analyses of differences in burden on the basis of age suggest that while most incident sequelae were higher in older adults; burdens of hyperlipidemia, chest pain, sleep disorders, headache, obesity, mood disorders, cough, and smell problems were higher in people younger than 60 years. Smaller differences of burden on the basis of race were observed where burden of acute kidney injury, diabetes mellitus, chest pain, cough, substance abuse, thromboembolism, headache, and tachycardia were slightly higher in Black persons; GERD and smell problems were higher in white persons. Burden of several sequelae in the respiratory system (shortness of breath and cough), cardiovascular system (chest pain, and arrhythmia), neurologic system (headache and smell problems), and dermatologic disorders (hair loss, and skin rash) were higher in females. Nearly all sequelae were more pronounced in persons with higher burden of baseline comorbidities (Fig. 4 and Supplementary Table 6).

### Burden of individual sequelae by demographic and health characteristics according to care setting of the acute infection

**Fig. 5:**
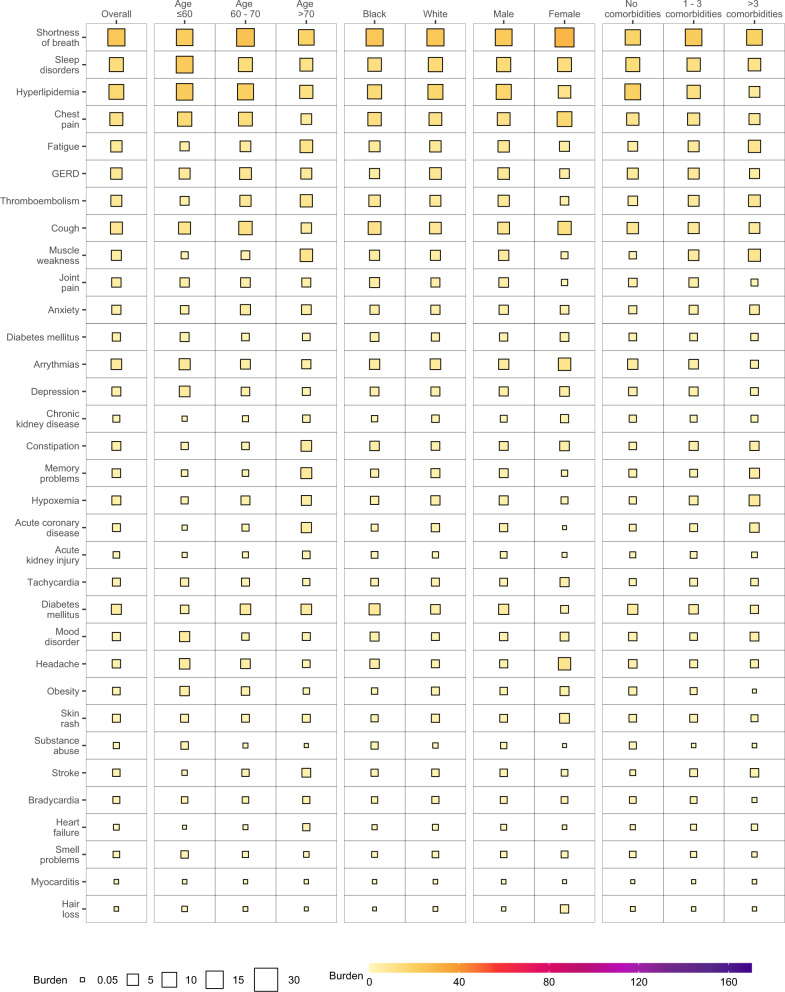
Burden of post-acute sequelae of COVID-19 in the overall cohort and by age, race, sex, and health status in non-hospitalized COVID-19. Estimates of burdens per 1000 COVID-19 patients at 6 months are presented. The size of the square represents the burden within each care setting. The intensity of color from light yellow to deep purple represents the range of burdens across care settings. Models adjusted for age, race, sex, receipt of long-term care, Area Deprivation Index, number of outpatient encounters, number of hospital admissions, number of outpatient prescriptions, number of outpatient serum creatinine measurements, chronic lung disease, cancer, cardiovascular disease, cerebrovascular disease, dementia, diabetes mellitus, hypertension, hyperlipidemia, depression, anxiety, chronic kidney disease, hepatitis C and peripheral artery disease, overweight, obesity, smoking status, Charlson Comorbidity Index, US geographic region, total number of beds, number of COVID-19 tests administered, COVID-19 positivity rate, and average hospital bed occupancy during the week of participant enrollment when appropriated.

**Fig. 6:**
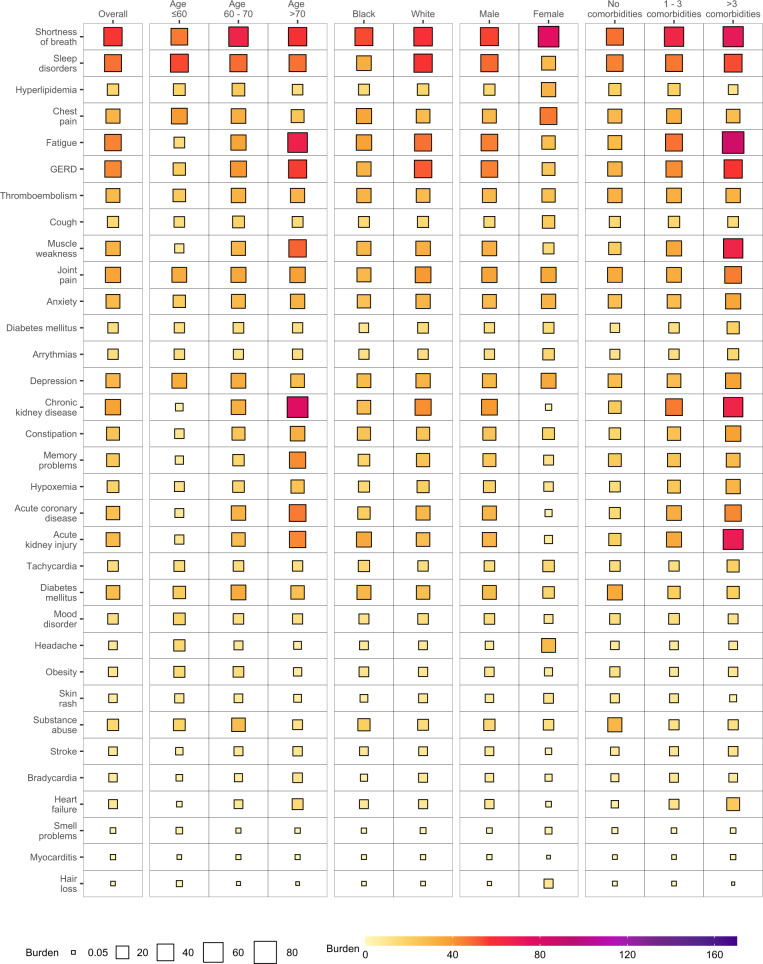
Burden of post-acute sequelae of COVID-19 in the overall cohort and by age, race, sex, and health status in hospitalized COVID-19. Estimates of burdens per 1000 COVID-19 patients at 6 months are presented. The size of the square represents the burden within each care setting. The intensity of color from light yellow to deep purple represents the range of burdens across care settings. Models adjusted for age, race, sex, receipt of long-term care, Area Deprivation Index, number of outpatient encounters, number of hospital admissions, number of outpatient prescriptions, number of outpatient serum creatinine measurements, chronic lung disease, cancer, cardiovascular disease, cerebrovascular disease, dementia, diabetes mellitus, hypertension, hyperlipidemia, depression, anxiety, chronic kidney disease, hepatitis C and peripheral artery disease, overweight, obesity, smoking status, Charlson Comorbidity Index, US geographic region, total number of beds, number of COVID-19 tests administered, COVID-19 positivity rate, and average hospital bed occupancy during the week of participant enrollment when appropriated.

**Fig. 7:**
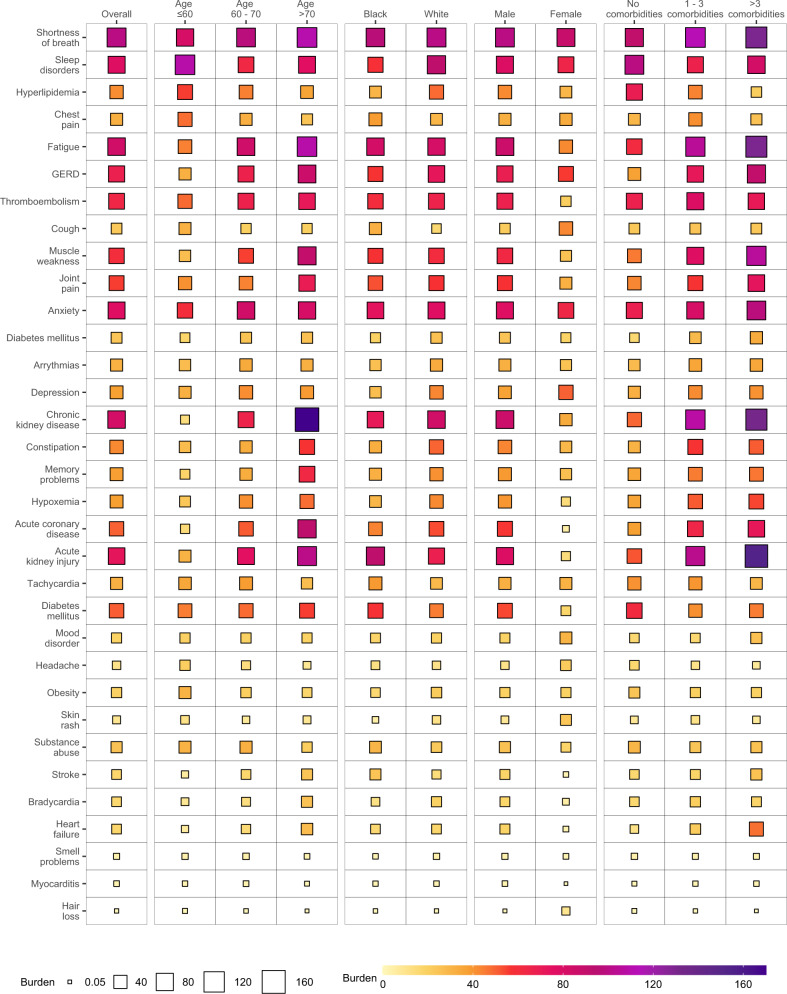
Burden of post-acute sequelae of COVID-19 in the overall cohort and by age, race, sex, and health status in COVID-19 patients admitted to intensive care. Estimates of burdens per 1000 COVID-19 patients at 6 months are presented. The size of the square represents the burden within each care setting. The intensity of color from light yellow to deep purple represents the range of burdens across care settings. Models adjusted for age, race, sex, receipt of long-term care, Area Deprivation Index, number of outpatient encounters, number of hospital admissions, number of outpatient prescriptions, number of outpatient serum creatinine measurements, chronic lung disease, cancer, cardiovascular disease, cerebrovascular disease, dementia, diabetes mellitus, hypertension, hyperlipidemia, depression, anxiety, chronic kidney disease, hepatitis C and peripheral artery disease, overweight, obesity, smoking status, Charlson Comorbidity Index, US geographic region, total number of beds, number of COVID-19 tests administered, COVID-19 positivity rate, and average hospital bed occupancy during the week of participant enrollment when appropriated.

In the overall cohort, burden of individual sequelae increased according to the care setting of the acute infection from non-hospitalized, hospitalized, and those who were admitted to intensive care (Figs. 5–7 and Supplementary Tables 7–9). In each subgroup based on age, race, sex, and baseline health status. The burden for each sequela increased as the intensity of care setting of the acute infection increased (non-hospitalized, hospitalized, admitted to intensive care).

### Risk and burden of individual sequelae after 12 weeks of the infection

To test whether the risk of post-acute sequelae persisted beyond the first 12 weeks of the infection, we conducted analyses where we defined post-acute sequelae as incident clinical manifestations in excess of the non-infected controls that occurred after 12 weeks (84 days) from the COVID-19-positive test. In adjusted analyses, the burden of PASC in the overall cohort was 59.36 (57.72, 60.97) per 1000 persons at 6 months (Supplementary Table 10). The burden of PASC increased according to care setting of the acute infection and was 40.60 (38.83, 42.26), 158.31 (152.92, 164.30), and 227.18 (215.65, 238.46) per 1000 persons at 6 months among non-hospitalized, hospitalized, and those who were admitted to intensive care during the first 30 days of infection, respectively (Supplementary Table 10). All individual post-acute sequelae exhibited increased risk and excess burden in COVID-19 compared to the controls. Risks and associated burdens for each sequela are provided in Supplementary Fig. 1 and Supplementary Table 11.

### Negative controls

Testing of risks of accidental injuries and neoplasms as negative outcome controls in people with COVID-19 (the overall COVID-19 cohort) yielded non-significant association between COVID-19 exposure and the risks of accidental injuries and neoplasms (Hazard ratio: 1.04 (0.84, 1.29) and 1.01 (0.88, 1.17), respectively) (Supplementary Table 10). The examination of negative outcome controls in groups according to care setting in non-hospitalized, hospitalized, and admitted to intensive care for COVID-19 also yielded non-significant associations—the results that were consistent with a priori expectations (Supplementary Table 12).

## Discussion

In this work, we estimate that PASC—defined as at least one sequela in excess of a non-infected control group—was 73.43 (72.10, 74.72) per 1000 persons at 6 months. The burden of PASC increased as a function of the severity of the acute infection as proxied by the care setting and was 44.51 (43.09, 45.85), 217.08 (212.43, 222.23), and 360.16 (350.53, 369.38) per 1000 persons at 6 months among non-hospitalized, hospitalized, and those who were admitted to intensive care during the first 30 days of infection, respectively. PASC is a multifaceted non-monolithic entity; some of its sequelae were more pronounced in younger individuals, and some were more pronounced in older adults. The same picture was evident in analyses across race groups (white and Black participants), and sex (males and females). The burden of individual sequela was consistently higher in people with poorer baseline health status and increased in a graded fashion according to intensity of the care setting of the acute infection. The constellation of findings shows that among the 30-day survivors of COVID-19, the burden of PASC is substantial (7%); PASC is not a monolithic entity with sequelae that are differentially expressed in various population groups. In sum, our results provide estimates of the burden for overall PASC; we additionally provide estimates of individual sequelae in various population groups by age, race, sex, and baseline health. Collectively, these results may be useful in informing health systems capacity planning and care strategies of people with PASC.

Here we estimate that the overall burden of PASC (long covid) beyond the first 30 days of illness is around 7%, (4.4%, 21.7%, and 36.5% among non-hospitalized, hospitalized, and those admitted to intensive care, respectively). The estimates were slightly lower but remained substantial using an alternative definition accounting for burden beyond first 12 weeks of illness (6% in overall cohort, and 4.1%, 15.8%, 22.7% in non-hospitalized, hospitalized, and those admitted to intensive care, respectively).

To date, most studies on long covid did not include a control group or simply focused mostly on people who were hospitalized; fewer studies reported burden of long covid in non-hospitalized patients with COVID-19^14–16^. Nevertheless, the evidence from the totality of studies accumulated thus far generally places the burden of long covid at around 4–10%^14–17^. Most importantly, it is now compellingly evident that while burden of PASC (or long covid) is most pronounced in people with poorer baseline health and those who had severe acute infection, the burden of PASC (or long covid) is substantial (and non-trivial) among non-hospitalized individuals (who represent the majority of people with COVID-19)—this potentially may translate into millions of people affected in the US and globally.

The implications of our results are clear. As the number of COVID-19 cases continues to climb across the globe, health systems face the dual challenge of coping with surges in acute infections, and caring for COVID-19 survivors (now accounting for more than 2% of the global population and growing) who will also likely require substantial care to mitigate permanent health loss. This will place additional demand on already strained health systems. Governments and health systems around the globe should be actively devising plans to address the tide of COVID-19 survivors in need of post-COVID-19 care. Our estimates of the burden of PASC (around 7% of infected people) and burden of 33 individual sequelae by age, race, sex, and baseline health status may help inform capacity planning and optimal composition of multidisciplinary post-COVID-19 clinics^11^.

Estimates of the burden of individual sequelae by age, race, sex, and baseline health status suggest a more nuanced picture in that the burden of some sequelae was more pronounced in younger adults (e.g., sleep disorders, headache, mood disorders, and smell problems), Black participants (e.g., new onset diabetes mellitus, chest pain, substance abuse, thromboembolism, headache, and tachycardia), and females (e.g., chest pain, arrhythmia, headache, smell problems, hair loss, and skin rash). These estimates provide insights into the long-term health consequences of COVID-19 and suggest that PASC is a complex multifaceted non-monolithic entity that may manifest differently in various population groups. These findings may also have implications on how we define and study the disease. A nuanced definition that considers the heterogeneity of key sequelae by demographic group will be needed. Future work should also focus on understanding whether subtypes or different phenotypes of long covid exist. A better understanding of the putative various phenotypes of long covid, their underlying biologic mechanisms, epidemiologic drivers, and associated clinical outcomes is needed to inform treatment strategies of people with long covid.

Here we use the term “PASC” to denote the consequences of post-covid in excess of what can be ascertained in the control group; while this definition is epidemiologically useful to estimate the burden of disease, a clinical definition is needed to guide the diagnosis and treatment^14^. Other terms used in the literature include “long COVID-19”, “post-COVID-19 syndrome”, and “post-acute COVID-19 syndrome”, and people with symptoms and clinical manifestations beyond the acute phase have been referred to in the lay vernacular as “long haulers”^3,14^. We recognize that this matter is subject to intense research by the scientific community and patient groups with lived experience of post COVID-19 who hold a broad range of views regarding these terminologies^13,14,18,19^. Identification of scientifically accurate definitions and culturally sensitive terms to describe the illness beyond the acute phase will be an important step not only to standardize scientific communications globally but also to support clear and consistent public health messaging about the long-term consequences of COVID-19^14^.

The study has several strengths. We used the vast national electronic health care databases of the US Department of Veterans Affairs—the largest nationally integrated healthcare delivery system in the US—to estimate burden of overall PASC and its individual sequelae by age, race, sex, and baseline health status. We comprehensively examined 33 sequelae which were defined based on integrated data from multiple sources including diagnostic codes, medications, and laboratory test results. The simultaneous examination of incident sequelae in the same analytic framework allows the comparative evaluation of risks and burdens of these conditions—providing health care providers, health system planners, public health officials, and the public at large with a priority list of the post-acute clinical conditions encountered in COVID-19 survivors. For each outcome examined, we built a cohort free of the related outcome at baseline to identify the risk of incident outcome during the follow-up—this approach allows the identification of incident clinical manifestations and abnormalities following COVID-19 infection. While we conducted survival analyses to estimate the risk of each outcome examined, we—for each outcome—also estimated the excess burden per 1000 persons due to COVID-19; this measure of risk on the absolute scale also considers the baseline risk and provides a more meaningful estimate of potential harm and can be more effectively communicated to the wider public than measures of relative risks (e.g., hazard ratio).

This study has several limitations. Our approach does not provide mechanistic insights into PASC nor does it delineate the sequelae that are direct or indirect consequences of the SARS-CoV-2 infection. Because of the predominantly male composition of the VA population, our findings may not identify clinical manifestations of post-acute COVID-19 that may be differentially much more pronounced in females and either non-expressed or rare in males. Our study included VHA users, and the results may not be representative of all US veterans. Our study did not include people who may have had COVID-19 but did not have a positive test for it; these individuals may have been included in the control arm, and if they exhibited sequelae related to COVID-19, it would have resulted in underestimation of risk. However, this is unlikely to be present in a large scale since testing constraints were present at the VA for only a brief period in March 2020 during the early phase of the pandemic. Our estimates of risks and associated burdens did not include undiagnosed conditions (i.e., people who may have suffered from some post-covid sequelae but did not seek care and their sequelae were not yet diagnosed). Finally, COVID-19 patients were enrolled in our cohorts from March 01, 2020 to March 15, 2021 and followed until May 01, 2021; as the COVID-19 global pandemic continues to evolve, and as treatment strategies improve, new variants of the virus emerge, and vaccine availability increases, it is likely that the epidemiology, short-term, and long-term outcomes of COVID-19 will likely also change over time^20^.

In conclusion, we estimate that the burden of PASC is around 7%; while the burden of PASC increased according to the severity of the care setting of the acute COVID-19 infection, it was not trivial (4.4%) among those who were not hospitalized for acute COVID-19. Our results also show that PASC is not monolithic; the burden of its individual components may be differentially expressed in various population groups. Together, the estimates provided here suggest that the toll of morbidity of COVID-19 extends well beyond the acute phase of the disease. While optimism is rising that—as vaccine availability increases—the pandemic may soon be behind us, the focus on the immediate health effects of COVID-19 allows visibility of the tip of the iceberg. The long-term consequences of COVID-19—as evidenced in our work—are substantial and will reverberate for a long time after the surges in acute infections abate. Long covid (or as we refer to it in this work PASC) is a complex multifaceted non-monolithic post-viral syndrome; it demands greater attention and a coordinated long-term global response strategy.

## Methods

### Setting

The study utilized the VA electronic health care databases. The VA provides health care to US Veterans and operates the largest national integrated healthcare system in the United States with 1255 health care facilities, including 170 VA medical centers and 1074 outpatient sites located across the United States. Veterans enrolled have access to the Department of Veterans Affairs comprehensive medical benefits package including inpatient hospital care; outpatient services; preventive, primary, and specialty care; prescriptions; mental healthcare; home healthcare; geriatric and extended care; medical equipment; and prosthetics. VA electronic health care databases are updated daily.

### Cohort

US Veterans who had at least one outpatient or inpatient encounter with the VHA between January 01, 2019 and December 31, 2019 were selected (*n* = 5,808,018). Among those alive on March 01, 2020 (*n* = 5,606,309), the COVID-19 group consisted of those with a COVID-19-positive test between March 01, 2020 and March 15, 2021 (*n* = 191,958). We then selected those who were alive on the 30th day after their first positive test (*n* = 181,384). COVID-19-positive patients were separated into 3 groups based on the care received during the 30 days after the positive test: (1) non-hospitalized (*n* = 155,987); (2) hospitalized (*n* = 19,359); (3) received intensive care (*n* = 6038). To generate a comparison group without COVID-19, we selected 4,534,600 participants who did not have a COVID-19-positive test, and randomly assigned every 25 of them the same cohort enrollment date as one participant in the COVID-19 group. We then further selected those who were alive 30 days after the enrollment date (VHA group *n* = 4,397,509). Participants were followed until May 01, 2021 (Supplementary Fig. 2).

### Data sources

Data used in this study was collected from the VA Corporate Data Warehouse (CDW)^21–26^. The CDW Outpatient Encounters and Inpatient Encounters domains provided information related to diagnoses, procedures, and hospitalization records^27^. The CDW Outpatient Pharmacy domain and CDW Bar Code Medication Administration domain were used to collect prescription information. CDW Patient domain was used to collect demographic information. The CDW Laboratory Results domain was used to collect laboratory test information, and the COVID-19 Shared Data Resource was used to collect COVID-19 tests. The Area Deprivation Index (ADI), a composite measure of income, education, employment, and housing was obtained from the University of Wisconsin^28^.

### Negative outcome controls

The use of negative controls in observational studies may help detect the presence of both suspected and unsuspected spurious biases; the application of negative controls will test if shared biases in outcome ascertainment, residual confounding, analytic approach, or other latent biases might have influenced the results^29,30^. Here we followed the approach outlined by Lipsitch and collaborators to test accidental injuries and neoplasms as negative outcome controls^29^, where, based on current knowledge, we would expect no association between COVID-19 infection and these two negative outcome controls.

### Post-acute sequelae of COVID-19

We examined a set of 33 post-acute COVID-19 outcomes; these outcomes were selected based on prior studies^1,31^, review of the literature^2,3^, and the most recent US National Institute of Health workshop on PASC. Outcomes were defined based on ICD10 codes recorded from inpatient or outpatient encounters, medication records, or laboratory tests when appropriate using definitions validated for use with electronic health records^26,31–41^. Detailed definitions of the outcomes are presented in Supplementary Table 13. Cardiovascular outcomes included acute coronary disease, arrhythmia, bradycardia, chest pain, heart failure, myocarditis, and tachycardia; coagulation outcomes included thromboembolism; dermatologic outcomes included hair loss and skin rash; endocrine outcomes included diabetes mellitus, hyperlipidemia, and obesity; gastrointestinal outcomes included constipation, diarrhea, and GERD; general outcomes included fatigue; kidney outcomes included acute kidney injury and chronic kidney disease; mental health outcomes included anxiety, depression, mood disorder, sleep disorder, and substance abuse; musculoskeletal outcomes included joint pain and muscle weakness; neurologic outcomes included headache, memory problems, smell problems, and stroke; and pulmonary outcomes included cough, hypoxemia, and shortness of breath. Occurrence of incident clinical manifestation was defined as the occurrence of a manifestation that did not occur within past 1 year before cohort enrollment. PASC was defined as the presence of at least one incident clinical manifestation in excess of the non-infected controls.

### Covariates

Covariates for analyses included age, race (white, Black, and other; race categorization was based on self-reports), sex, receipt of long-term care, Area Deprivation Index based on residential addresses and proxies of healthcare utilization, such as number of outpatient encounters, number of hospital admissions, number of outpatient prescriptions, and number of outpatient serum creatinine measurements in the year before enrollment. We also included comorbidities, such as chronic lung disease, cancer, cardiovascular disease, cerebrovascular disease, dementia, diabetes mellitus, hypertension, hyperlipidemia, depression, anxiety, chronic kidney disease, and hepatitis C and peripheral artery disease. In addition, covariates included overweight, obesity, smoking status (never, former, and current) and the Charlson Comorbidity Index were also adjusted for. We also adjusted for US geographic region (West, Mid-west, South and Northeast) where the care was received, and additional health system characteristics including total number of beds, number of COVID-19 tests administered, COVID-19 positivity rate, and average hospital bed occupancy during the week of participant enrollment.

### Statistical analyses

Characteristics of the VHA users without COVID-19, and those with COVID-19 according to care setting of the acute infection (non-hospitalized, hospitalized, and admitted to intensive care) were described.

Excess burden of PASC, defined as having at least one sequela in excess of VHA users without COVID-19, was estimated using Poisson regression. The excess burdens of having 1, 2, to 33 sequelae at 6 months, as well as the total excess burden of PASC, were estimated in the overall cohort and by care setting of the acute infection.

We then estimated the excess burden of incident individual sequela. For each outcome examined, we built a cohort of participants without a history of the outcome. Cox models adjusting for covariates were used to estimate the hazard ratio of each COVID-19 care setting compared to VHA users, and the survival probability for the 4 groups at 6 months. Cause specific hazard models were used where occurrence of death was considered as competing risk during the analyses. Excess burden per 1000 patients at 6 months were computed as the difference in survival probability between each COVID-19 care setting and the VHA users group. Burden of outcomes in the overall COVID-19 population was computed as the weighted sum of the burden of the three care settings based on the proportion of COVID-19 patients in each care setting. Analyses were also conducted to estimate the excess burden within subgroups by age, race, sex, and baseline health status. Burden differences between subgroups of age ≤60 and >70, Black and white, female and male, and 0 and >3 comorbidity score were then estimated.

To test whether the risk of post-acute sequelae persisted beyond the first 12 weeks of the infection, we conducted additional analyses to estimate the burden of overall PASC and burden of each incident sequela, where we defined post-acute sequelae as incident clinical manifestations in excess of the non-infected controls that occurred after 12 weeks (84 days) from the COVID-19-positive test.

All analyses were done using SAS Enterprise Guide version 7.1 (SAS Institute, Cary, NC). Data visualizations were performed in R 4.0.3 (R Foundation for Statistical Computing, Vienna, Austria). The study and informed consent waivers were approved by the Institutional Review Board of the Department of Veterans Affairs St. Louis Health Care System, St. Louis, MO. The study was reported following the Strengthening the Reporting of Observational Studies in Epidemiology (STROBE) guidelines for reporting observational studies. This research project was reviewed and approved by the Institutional Review Board of the Department of Veterans Affairs Saint Louis Health Care System.

### Reporting summary

Further information on research design is available in the Nature Research Reporting Summary linked to this article.

## Supplementary information

Supplementary information.

Reporting summary.

## Data Availability

The data that support the findings of this study are available from the VA. VA data are made freely available to researchers behind the VA firewall with an approved VA study protocol. More information is available at https://www.virec.research.va.gov or by contacting the VA Information Resource Center (VIReC) at VIReC@va.gov.

## Code availability

SAS codes are available at https://github.com/yxie618/longPASC and at 10.5281/zenodo.5555241^42^.
