# Supplementary material for: Burdens of post-acute sequelae of COVID-19 by severity of acute infection, demographics and health status
Source: Nat Commun. 2021 Nov 12;12:6571. doi: 10.1038/s41467-021-26513-3 (PMC8589966; doi:10.1038/s41467-021-26513-3)
Supplement: Supplementary file 1 — Supplementary information. [file 41467_2021_26513_MOESM1_ESM.pdf]

Supplementary Information

Burdens of post-acute sequelae of COVID-19 by severity of acute infection, demographics and health status

Yan Xie; Benjamin Bowe; and Ziyad Al-Aly

Supplementary figures:

Supplementary Figure 1: Risks and burdens of Post-Acute Sequelae of SARS-CoV-2 infection (PASC) after 12 weeks. Risks and burdens and 95% confidence intervals at 6-months were estimated at 6 months. Estimates of burdens per 1000 COVID-19 patients at 6-months are presented.

Supplementary Figure 2: A schematic of cohort design.

Supplementary tables:

Supplementary Table 1: Unadjusted number and percentage of incident clinical manifestation in 1) users of Veterans Health Administration (VHA) without COVID-19, 2) COVID-19 patients, 3) non-hospitalized COVID-19 patients, 4) hospitalized COVID-19 patients, and 5) COVID-19 patients admitted to intensive care.

Supplementary Table 2: Risks and burdens of individual sequela in 30-day survivors of COVID-19.

Supplementary Table 3: Unadjusted overall burden of Post-Acute Sequelae of SARS-CoV-2 infection (PASC) per 1000 persons at 6 months in the overall cohort and across care setting (non-hospitalized, hospitalized, and admitted to intensive care during the acute phase of the infection).

Supplementary Table 4: Adjusted burden of individual clinical manifestations per 1000 persons at 6 months in 1) users of Veterans Health Administration (VHA) without COVID-19, 2) Overall COVID-19 patients, 3) non-hospitalized COVID-19 patients, 4) hospitalized COVID-19 patients, and 5) COVID-19 patients admitted to intensive care.

Supplementary Table 5: Burden of Post-acute Sequelae of COVID-19 individual sequelae in the overall cohort and by age, race, sex, and health status.

Supplementary Table 6: Differences in burden of individual Post-acute Sequelae of COVID-19 by age, race, sex, and health status in the overall cohort.

Supplementary Table 7: Burden of Post-acute Sequelae of COVID-19 individual sequelae in the overall cohort and by age, race, sex, and health status in non-hospitalized COVID-19.

Supplementary Table 8: Burden of Post-acute Sequelae of COVID-19 individual sequelae in the overall cohort and by age, race, sex, and health status in hospitalized COVID-19.

Supplementary Table 9: Burden of Post-acute Sequelae of COVID-19 individual sequelae in the overall cohort and by age, race, sex, and health status in COVID-19 admitted to intensive care.

Supplementary Table 10: Overall burden of Post-Acute Sequelae of SARS-CoV-2 infection (PASC) after 12 weeks per 1000 persons at 6 months in the overall cohort and across care setting (non-hospitalized, hospitalized, and admitted to intensive care during the acute phase of the infection).

Supplementary Table 11: Risks and burdens of individual sequela after 12 weeks infection of COVID-19.

Supplementary Table 12: Results of negative outcome controls in people with COVID-19, and in non-hospitalized, hospitalized, and admitted to intensive care for COVID-19.

Supplementary Table 13: Definition of each Post-Acute Sequela of SARS-CoV-2 infection

Supplementary Figure 1: Risks and burdens of Post-Acute Sequelae of SARS-CoV-2 infection (PASC) after 12 weeks. Hazard ratio and 95% confidence intervals were are presented in left panel and burdens per 1000 COVID-19 patients at 6-months are presented in right panel

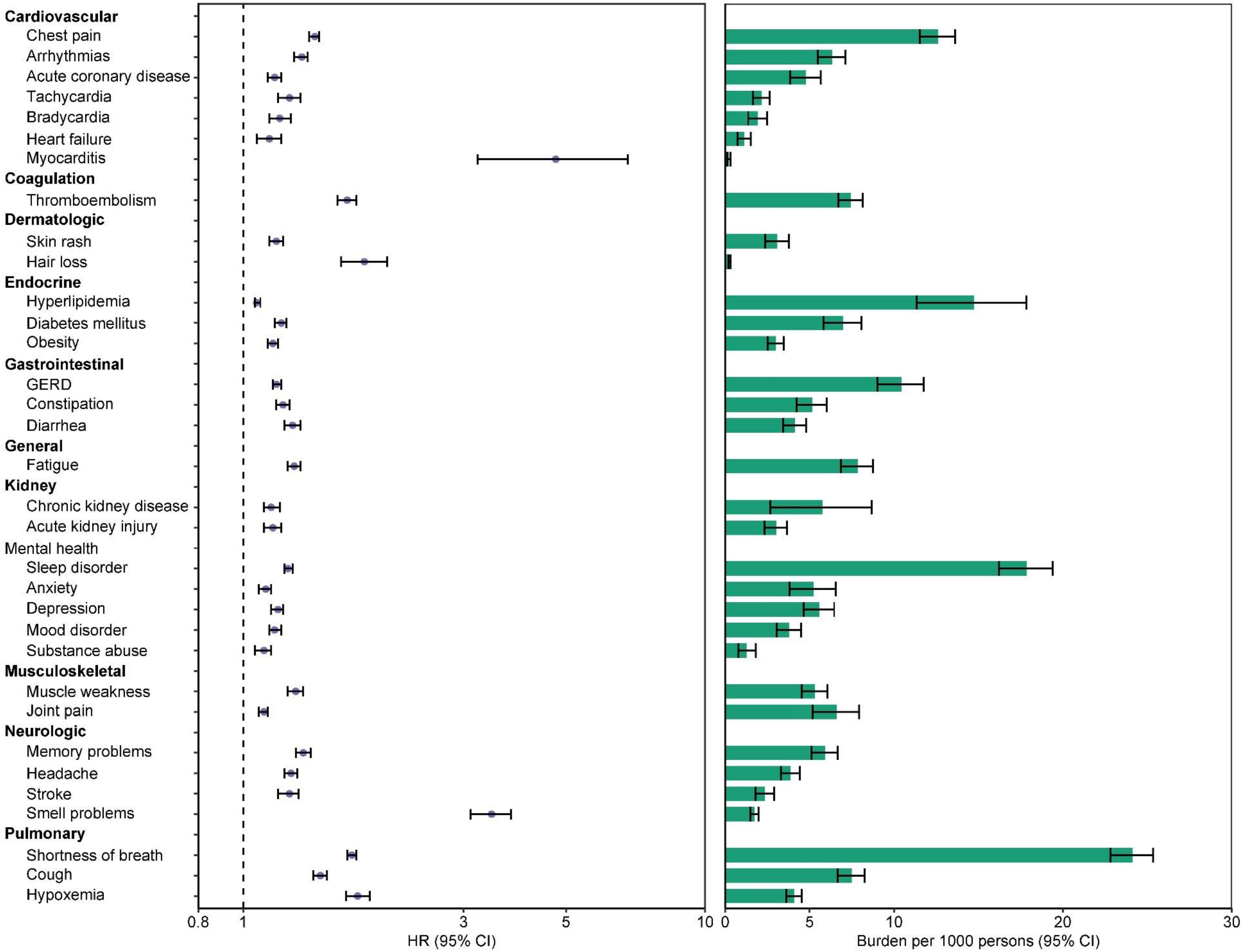

Supplementary Figure 2: A schematic of cohort design

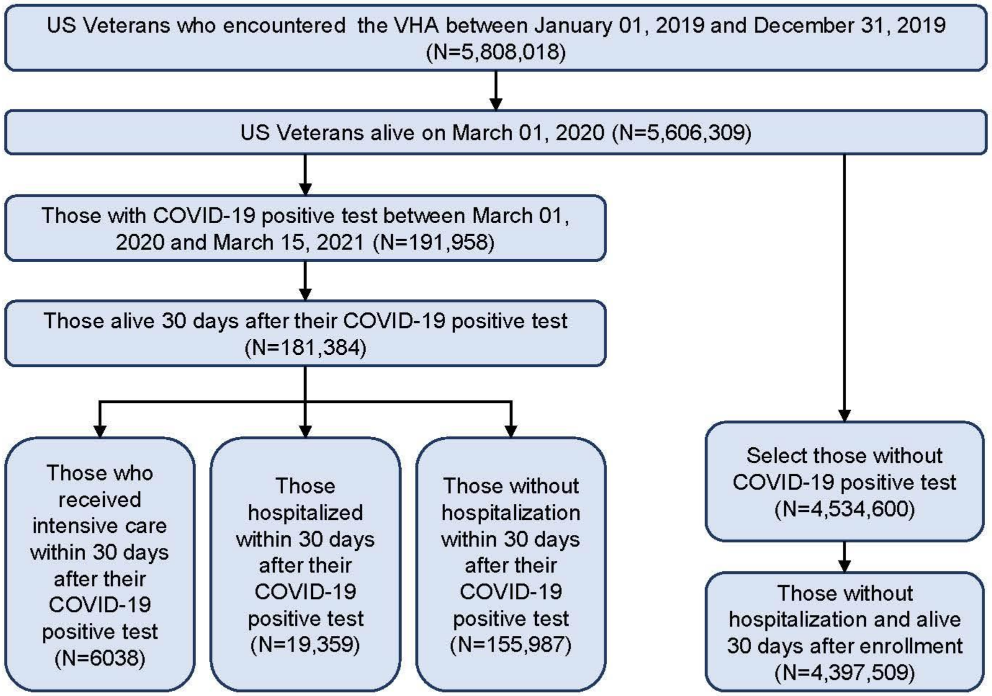

Supplementary Table 1: Unadjusted number and percentage of incident clinical manifestations during the follow up in 1) users of Veterans Health Administration (VHA) without COVID-19, 2) Overall COVID-19 patients, 3) non-hospitalized COVID-19 patients, 4) hospitalized COVID-19 patients, and 5) COVID-19 patients admitted to intensive care

Organ	Clinical manifestation	VHA users<br><br>Unadjusted number of events (%)	Overall COVID-19<br><br>Unadjusted number of events (%)	Non-hospitalized COVID-19 Unadjusted number of events (%)	Hospitalized COVID-19<br><br>Unadjusted number of events (%)	COVID-19 admitted to intensive care Unadjusted number of events (%)
Cardiovascular	Acute coronary disease	60217 (1.54)	3694 (2.36)	2332 (1.7)	910 (6.14)	452 (10.01)
	Arrythmias	33623 (0.78)	3073 (1.77)	2217 (1.48)	561 (3.12)	295 (5.41)
	Bradycardia	14729 (0.34)	1297 (0.73)	787 (0.51)	327 (1.76)	183 (3.16)
	Chest pain	54552 (1.29)	5270 (3.16)	3897 (2.69)	1027 (6.22)	346 (6.67)
	Heart failure	11862 (0.27)	1464 (0.82)	643 (0.42)	532 (2.91)	289 (5.17)
	Myocarditis	125 (0)	73 (0.04)	30 (0.02)	26 (0.13)	17 (0.28)
	Tachycardia	12497 (0.29)	1710 (0.96)	885 (0.57)	511 (2.75)	314 (5.45)
Coagulation	Thromboembolism	15795 (0.36)	2771 (1.57)	1423 (0.93)	821 (4.48)	527 (9.23)
Dermatologic	Hair loss	2050 (0.05)	244 (0.13)	204 (0.13)	31 (0.16)	9 (0.15)
	Skin rash	33188 (0.77)	2333 (1.33)	1790 (1.19)	402 (2.18)	141 (2.45)
Endocrine	Diabetes mellitus	61533 (1.83)	3570 (2.91)	2709 (2.48)	592 (5.64)	269 (8.91)
	Hyperlipidemia	254134 (7.84)	12959 (10.82)	11056 (10.74)	1377 (10.65)	526 (13.38)
	Obesity	78868 (1.98)	5496 (3.58)	4327 (3.26)	793 (5)	376 (7.71)
Gastrointestinal	Constipation	38889 (0.91)	3658 (2.13)	2187 (1.46)	1019 (5.91)	452 (8.4)
	Diarrhea	25255 (0.58)	2363 (1.36)	1534 (1.02)	563 (3.13)	266 (4.69)
	GERD	110376 (3)	6965 (4.9)	5182 (4.19)	1272 (8.98)	511 (11.68)
General	Fatigue	39788 (0.93)	4962 (2.89)	2631 (1.76)	1553 (9.3)	778 (14.88)
Kidney	Acute kidney injury	23875 (0.55)	3307 (1.89)	1237 (0.81)	1257 (7.31)	813 (15.49)
	Chronic kidney disease	131914 (3.47)	5662 (3.82)	4486 (3.41)	876 (6.73)	300 (7.70)
Mental health	Anxiety	46434 (1.15)	4239 (2.67)	2625 (1.9)	985 (6.29)	629 (12.77)
	Depression	60262 (1.47)	4531 (2.8)	3167 (2.26)	999 (6.08)	365 (7.01)
	Mood disorder	56112 (1.4)	3708 (2.34)	2886 (2.11)	600 (3.58)	222 (4.19)
	Sleep disorder	135989 (4)	8995 (7.44)	7037 (6.71)	1417 (11.45)	541 (14.59)
	Substance abuse	85234 (2.21)	4134 (2.65)	3165 (2.33)	689 (4.47)	280 (5.7)
Musculoskeletal	Joint pain	108830 (2.84)	6873 (4.67)	5097 (3.96)	1273 (9.01)	503 (11.51)
	Muscle weakness	26763 (0.62)	3703 (2.12)	1817 (1.19)	1249 (7.19)	637 (11.79)

Neurologic	Headache	41186 (0.98)	3161 (1.89)	2573 (1.79)	429 (2.4)	159 (2.84)
	Memory problems	32078 (0.75)	2769 (1.63)	1564 (1.05)	821 (4.92)	384 (7.23)
	Smell problems	1691 (0.04)	356 (0.2)	279 (0.18)	49 (0.25)	28 (0.46)
	Stroke	16888 (0.39)	1456 (0.83)	880 (0.58)	380 (2.1)	196 (3.44)
Pulmonary	Cough	28088 (0.66)	3041 (1.83)	2290 (1.59)	536 (3.13)	215 (4.04)
	Hypoxemia	9067 (0.21)	2017 (1.13)	890 (0.58)	700 (3.72)	427 (7.37)
	Shortness of breath	58454 (1.38)	7627 (4.58)	5176 (3.56)	1653 (10.2)	798 (16.03)
Unadjusted number and percentage of incident clinical manifestation during the follow up are obtained within participants without the occurrence of event within one year before enrollment.						

Supplementary Table 2: Risks and burdens of individual sequela in 30-day survivors of COVID-19

Organ	Sequelae	Hazard Ratio (95% CI)	Burden per 1000 persons at 6 months (95% CI)
Cardiovascular	Chest pain	1.68 (1.62, 1.73)	13.84 (12.86, 14.76)
	Arrythmias	1.59 (1.52, 1.66)	7.85 (7.08, 8.58)
	Acute coronary disease	1.30 (1.25, 1.35)	6.18 (5.37, 6.96)
	Tachycardia	1.77 (1.65, 1.89)	4.69 (4.18, 5.21)
	Bradycardia	1.42 (1.32, 1.51)	2.63 (2.14, 3.1)
	Heart failure	1.40 (1.30, 1.5)	2.02 (1.67, 2.37)
	Myocarditis	7.91 (5.27, 11.53)	0.32 (0.23, 0.45)
Coagulation	Thromboembolism	2.59 (2.46, 2.72)	11.35 (10.59, 12.12)
Dermatologic	Skin rash	1.27 (1.21, 1.33)	3.3 (2.67, 3.89)
	Hair loss	1.93 (1.54, 2.37)	0.22 (0.17, 0.29)
Endocrine	Hyperlipidemia	1.22 (1.19, 1.24)	17.09 (15.28, 18.79)
	Diabetes mellitus	1.39 (1.33, 1.44)	8.47 (7.46, 9.44)
	Obesity	1.28 (1.24, 1.32)	3.49 (3.06, 3.92)
Gastrointestinal	GERD	1.33 (1.3, 1.37)	13.25 (11.97, 14.49)
	Constipation	1.41 (1.35, 1.47)	7.13 (6.33, 7.9)
	Diarrhea	1.48 (1.41, 1.56)	4.68 (4.09, 5.27)
General	Fatigue	1.75 (1.68, 1.81)	13.59 (12.66, 14.49)
Kidney	Chronic kidney disease	1.18 (1.13, 1.23)	7.19 (5.78, 8.55)
	Acute kidney injury	1.41 (1.34, 1.48)	6.07 (5.46, 6.69)
Mental health	Sleep disorders	1.41 (1.37, 1.44)	19.51 (18.05, 20.92)
	Anxiety	1.47 (1.41, 1.53)	8.75 (7.96, 9.55)
	Depression	1.37 (1.32, 1.42)	7.61 (6.8, 8.42)
	Mood disorder	1.31 (1.26, 1.36)	4.57 (3.93, 5.21)
	Substance abuse	1.16 (1.12, 1.2)	3.24 (2.55, 3.92)
Musculoskeletal	Muscle weakness	1.82 (1.74, 1.9)	9.81 (9.05, 10.55)
	Joint pain	1.21 (1.18, 1.24)	8.92 (7.67, 10.1)
Neurologic	Memory problems	1.56 (1.48, 1.63)	6.46 (5.78, 7.13)
	Headache	1.39 (1.33, 1.44)	3.8 (3.32, 4.28)
	Stroke	1.5 (1.4, 1.59)	3.11 (2.6, 3.59)
	Smell problems	3.61 (3.15, 4.11)	1.34 (1.13, 1.56)
Pulmonary	Shortness of breath	2.23 (2.17, 2.29)	28.8 (27.55, 29.97)

	Cough	2.02 (1.93, 2.10)	10.24 (9.46, 10.98)
	Hypoxemia	2.83 (2.65, 3.01)	6.43 (5.94, 6.93)

Adjusted hazard ratio, adjusted burden per 1000 persons at 6 months of people with COVID-19 compared to users of the Veterans Health Administration. All users of the VHA served as the referent category. Outcomes were ascertained from day 30 after COVID-19 diagnosis until end of follow-up. For each outcome, cohort participants without history of the outcome in past one year were included in the analysis. Within each organ system, sequalae are ranked based on excess burden from high to low.

Supplementary Table 3: Unadjusted overall burden of Post-Acute Sequelae of SARS-CoV-2 infection (PASC) per 1000 persons at 6 months in the overall cohort and across care setting (non-hospitalized, hospitalized, and admitted to intensive care during the acute phase of the infection).

	COVID-19 group Unadjusted burden per 1000 persons at 6 months (95% CI)	Users of the VHA without COVID-19 Unadjusted burden per 1000 persons at 6 months (95% CI)	Burden associated with COVID-19† Unadjusted burden per 1000 persons at 6 months (95% CI)
Overall	237.38 (235.52, 239.37)	88.96 (88.69, 89.19)	148.44 (146.59, 150.41)
Non-hospitalized COVID-19*	184.52 (182.51, 186.63)	88.96 (88.69, 89.19)	95.57 (93.59, 97.69)
Hospitalized COVID-19*	518.56 (511.37, 525.09)	88.96 (88.69, 89.19)	429.61 (422.34, 436.24)
COVID-19 required ICU*	702.28 (691.52, 712.38)	88.96 (88.69, 89.19)	613.32 (602.67, 623.38)

*. Care settings during the first 30 days of infection.
†. Burden defined as having at least one sequela in excess of users of the Veterans Health Administration without COVID-19 after the first 30-days of infection. Burden was estimated based on Poisson regression.

Supplementary Table 4: Adjusted burden of individual clinical manifestations per 1000 persons at 6 months in 1) users of Veterans Health Administration (VHA) without COVID-19, 2) Overall COVID-19 patients, 3) non-hospitalized COVID-19 patients, 4) hospitalized COVID-19 patients, and 5) COVID-19 patients admitted to intensive care

Organ	Clinical manifestations	Without COVID-19 Adjusted burden per 1000 persons at 6 months (95% CI)	Overall COVID-19 Adjusted burden per 1000 persons at 6 months (95% CI)	Non-hospitalized COVID-19 Adjusted burden per 1000 persons at 6 months (95% CI)	Hospitalized COVID-19 Adjusted burden per 1000 persons at 6 months (95% CI)	COVID-19 admitted to intensive care Adjusted burden per 1000 persons at 6 months (95% CI)
Kidney	Acute kidney injury	9.60 (9.44, 9.76)	16.52 (15.85, 17.21)	11.08 (10.42, 11.72)	39.83 (37.48, 42.16)	88.42 (82.29, 94.76)
Mental health	Anxiety	15.38 (15.17, 15.59)	24.44 (23.64, 25.23)	19.72 (18.86, 20.54)	43.37 (40.49, 46.24)	92.84 (85.5, 100.47)
Cardiovascular	Bradycardia	5.66 (5.53, 5.79)	8.06 (7.57, 8.53)	7.25 (6.7, 7.79)	12.59 (11.17, 14.07)	23.04 (19.72, 26.76)
Cardiovascular	Chest pain	19.56 (19.31, 19.8)	33.59 (32.61, 34.51)	30.49 (29.4, 31.53)	48.12 (44.98, 51.23)	51.44 (45.95, 57.35)
Kidney	Chronic kidney disease	31.80 (31.57, 32.03)	39.63 (38.13, 41.11)	33.34 (31.77, 34.86)	71.13 (66.09, 76.17)	118.68 (107.37, 130.65)
Gastrointestinal	Constipation	14.94 (14.73, 15.14)	22.34 (21.54, 23.11)	18.75 (17.86, 19.61)	39.13 (36.58, 41.68)	58.37 (52.93, 64.13)
Pulmonary	Cough	9.77 (9.59, 9.94)	20.11 (19.33, 20.84)	18.66 (17.78, 19.51)	25.1 (22.83, 27.41)	31.36 (27.08, 36.14)
Mental health	Depression	18.33 (18.1, 18.57)	26.55 (25.73, 27.35)	22.37 (21.47, 23.22)	47.42 (44.28, 50.53)	55.09 (49.36, 61.26)
Gastrointestinal	Diarrhea	8.79 (8.63, 8.95)	13.61 (13.02, 14.2)	11.74 (11.07, 12.39)	21.58 (19.68, 23.52)	32.96 (28.94, 37.37)
Endocrine	Diabetes mellitus	21.49 (21.18, 21.8)	29.38 (28.37, 30.35)	27.36 (26.24, 28.43)	49.39 (45.27, 53.59)	75.63 (66.74, 85.24)
General	Fatigue	15.82 (15.61, 16.02)	29.26 (28.34, 30.16)	23.82 (22.81, 24.79)	60.45 (57.26, 63.6)	102.24 (95.04, 109.7)
Gastrointestinal	Gerd	39.08 (38.7, 39.46)	52.76 (51.48, 54)	47.44 (46.02, 48.79)	83.48 (78.75, 88.15)	107.67 (98.4, 117.36)
Dermatologic	Hair loss	0.14 (0.11, 0.16)	0.43 (0.37, 0.49)	0.25 (0.2, 0.31)	0.35 (0.19, 0.61)	0.3 (0.09, 0.96)
Neurologic	Headache	8.84 (8.67, 9.01)	13.19 (12.71, 13.67)	11.62 (11.07, 12.15)	15.43 (13.82, 17.1)	19.64 (16.51, 23.2)
Cardiovascular	Arrythmias	12.19 (12, 12.39)	20.13 (19.35, 20.86)	18.1 (17.23, 18.93)	24.8 (22.62, 27.01)	44.01 (38.96, 49.51)

Cardiovascular	Heart failure	4.12 (4.01, 4.22)	5.83 (5.48, 6.18)	4.92 (4.52, 5.32)	12.98 (11.81, 14.18)	22.48 (19.86, 25.35)
Endocrine	Hyperlipidemia	86.19 (85.51, 86.86)	103.99 (102.18, 105.69)	103.19 (101.12, 105.14)	102.01 (96.43, 107.56)	128.56 (117.62, 139.87)
Pulmonary	Hypoxemia	3 (2.91, 3.09)	9.3 (8.81, 9.8)	7.03 (6.52, 7.53)	21.86 (20.07, 23.67)	42.66 (38.55, 47.15)
Musculoskeletal	Joint pain	41.44 (41.07, 41.82)	50.57 (49.32, 51.75)	46.27 (44.87, 47.6)	77.27 (72.86, 81.63)	98.56 (90.02, 107.5)
Neurologic	Memory problems	10.4 (10.22, 10.57)	16.51 (15.83, 17.18)	14.16 (13.4, 14.89)	33.95 (31.5, 36.41)	49.44 (44.4, 54.81)
Cardiovascular	Acute coronary disease	18.92 (18.66, 19.17)	23.39 (22.58, 24.17)	21.86 (20.92, 22.75)	48.12 (44.9, 51.34)	75.24 (68.39, 82.48)
Mental health	Mood disorder	14 (13.78, 14.21)	19.07 (18.43, 19.71)	16.97 (16.26, 17.65)	27.61 (25.28, 29.97)	32.52 (28.15, 37.34)
Musculoskeletal	Muscle weakness	10.61 (10.44, 10.78)	20.21 (19.45, 20.95)	16.59 (15.76, 17.4)	42.92 (40.37, 45.45)	73.71 (67.93, 79.78)
Cardiovascular	Myocarditis	0.03 (0.03, 0.04)	0.36 (0.26, 0.49)	0.2 (0.13, 0.31)	1.06 (0.65, 1.69)	2.14 (1.21, 3.72)
Endocrine	Obesity	11.47 (11.22, 11.73)	15.56 (15.13, 15.99)	13.63 (13.16, 14.07)	20.92 (19.36, 22.48)	30.99 (27.79, 34.41)
Pulmonary	Shortness of breath	21.95 (21.7, 22.2)	50.51 (49.27, 51.68)	44.14 (42.77, 45.43)	76.83 (72.92, 80.69)	122.15 (113.71, 130.83)
Dermatologic	Skin rash	11.07 (10.89, 11.26)	14.54 (13.91, 15.14)	13.45 (12.74, 14.14)	17.61 (15.78, 19.5)	19.13 (15.9, 22.85)
Mental health	Sleep disorder	48.43 (47.99, 48.87)	68.81 (67.35, 70.22)	62.87 (61.24, 64.41)	97.22 (92.03, 102.33)	126.82 (116.29, 137.77)
Neurologic	Smell problems	0.48 (0.44, 0.52)	1.82 (1.61, 2.04)	1.67 (1.44, 1.92)	1.78 (1.27, 2.44)	3.69 (2.45, 5.49)
Neurologic	Stroke	5.98 (5.84, 6.11)	8.8 (8.29, 9.29)	8.11 (7.54, 8.68)	13.78 (12.33, 15.27)	24.13 (20.78, 27.86)
Mental health	Substance abuse	18.66 (18.41, 18.91)	21.66 (20.97, 22.34)	19.79 (19.03, 20.51)	34.85 (32.12, 37.62)	44.29 (39.07, 49.94)
Cardiovascular	Tachycardia	4.74 (4.62, 4.85)	9.48 (8.97, 10)	7.14 (6.6, 7.67)	18.15 (16.45, 19.91)	36.78 (32.6, 41.34)
Coagulation	Thromboembolism	6.41 (6.27, 6.54)	17.57 (16.81, 18.34)	14.11 (13.3, 14.9)	34.77 (32.18, 37.39)	73.81 (67.51, 80.56)
Clinical manifestations were ascertained from 30 days after infection until end of follow-up.						

Supplementary Table 5: Burden of Post-acute Sequelae of COVID-19 individual sequelae in the overall cohort and by age, race, sex, and health status

Organ	Sequelae	Overall	Age			Race	
			Age ≤ 60	Age >60 - ≤ 70	Age >70	Black	White
Pulmonary	Shortness of breath	28.80 (27.55, 29.97)	23.56 (21.94, 25.09)	32.41 (29.76, 35.02)	27.82 (25.81, 29.84)	30.13 (27.69, 32.53)	28.39 (26.86, 29.8)
Mental health	Sleep disorder	19.51 (18.05, 20.92)	25.07 (22.08, 27.85)	19.51 (16.12, 22.8)	19.38 (17.08, 21.71)	17.32 (14.52, 20.14)	20.48 (18.73, 22.16)
Endocrine	Hyperlipidemia	17.09 (15.28, 18.79)	21.85 (18.36, 25.1)	21.25 (16.86, 25.46)	13.32 (10.58, 16.06)	15.23 (11.98, 18.41)	17.64 (15.39, 19.73)
Cardiovascular	Chest pain	13.84 (12.86, 14.76)	16.55 (14.94, 18.09)	16.13 (13.88, 18.34)	9.91 (8.49, 11.34)	16.46 (14.39, 18.5)	13.07 (11.94, 14.14)
General	Fatigue	13.59 (12.66, 14.49)	5.40 (4.46, 6.3)	12.87 (11.05, 14.73)	22.79 (21.02, 24.6)	13.94 (12.13, 15.79)	13.29 (12.19, 14.36)
Gastrointestinal	GERD	13.25 (11.97, 14.49)	8.41 (6.64, 10.09)	14.46 (11.71, 17.2)	17.8 (15.58, 20.05)	11.04 (8.71, 13.42)	14.11 (12.5, 15.65)
Coagulation	Thromboembolism	11.35 (10.59, 12.12)	5.65 (4.84, 6.46)	12.55 (10.96, 14.22)	15.66 (14.31, 17.07)	13.32 (11.72, 15)	10.64 (9.76, 11.53)
Pulmonary	Cough	10.24 (9.46, 10.98)	9.87 (8.71, 10.97)	13.37 (11.6, 15.12)	8.13 (6.95, 9.33)	12.37 (10.76, 13.99)	9.37 (8.46, 10.24)
Musculoskeletal	Muscle weakness	9.81 (9.05, 10.55)	2.54 (1.9, 3.18)	8.45 (7.01, 9.97)	19.48 (17.89, 21.1)	10.89 (9.35, 12.48)	9.38 (8.49, 10.25)
Musculoskeletal	Joint pain	8.92 (7.67, 10.1)	6.52 (4.69, 8.27)	9.42 (6.64, 12.13)	11.47 (9.38, 13.58)	10.25 (7.67, 12.81)	8.42 (6.96, 9.81)
Mental health	Anxiety	8.75 (7.96, 9.55)	5.04 (3.84, 6.17)	11.03 (9.16, 12.98)	12.33 (10.96, 13.76)	9.83 (8.2, 11.55)	8.52 (7.6, 9.44)
Endocrine	Diabetes mellitus	8.47 (7.46, 9.44)	4.67 (3.56, 5.74)	10.46 (8.2, 12.76)	11.07 (9.17, 13.01)	11.31 (9.03, 13.6)	7.29 (6.18, 8.35)
Cardiovascular	Arrythmias	7.85 (7.08, 8.58)	8.06 (7, 9.08)	7 (5.48, 8.54)	6.81 (5.56, 8.09)	7.66 (6.21, 9.13)	7.83 (6.91, 8.72)
Mental health	Depression	7.61 (6.8, 8.42)	8.51 (6.93, 10.07)	8.19 (6.31, 10.14)	7.81 (6.53, 9.13)	7.93 (6.27, 9.65)	7.41 (6.47, 8.34)
Kidney	Chronic kidney disease	7.19 (5.78, 8.55)	0.87 (0.18, 1.57)	6.53 (4.02, 9.11)	19.24 (15.43, 23.14)	6.63 (4.74, 8.58)	7.52 (5.52, 9.43)
Gastrointestinal	Constipation	7.13 (6.33, 7.9)	2.77 (1.94, 3.57)	5.81 (4.25, 7.4)	12.51 (10.98, 14.06)	8.4 (6.73, 10.09)	6.67 (5.74, 7.56)
Neurologic	Memory problems	6.46 (5.78, 7.13)	1.91 (1.34, 2.47)	4.25 (3.08, 5.51)	14.63 (13.04, 16.26)	5.93 (4.74, 7.17)	6.86 (6, 7.69)
Pulmonary	Hypoxemia	6.43	2.72	6.89	10.25	5.92	6.84

		(5.94, 6.93)	(2.24, 3.22)	(5.94, 7.93)	(9.32, 11.24)	(5.02, 6.92)	(6.21, 7.47)
Cardiovascular	Acute coronary disease	6.18 (5.37, 6.96)	1.12 (0.53, 1.73)	6.84 (5.11, 8.64)	15.07 (13.06, 17.08)	5.63 (4.21, 7.11)	6.45 (5.38, 7.48)
Kidney	Acute kidney injury	6.07 (5.46, 6.69)	1.33 (0.81, 1.86)	6.36 (5.15, 7.69)	12.32 (11.12, 13.58)	9.15 (7.74, 10.66)	5.08 (4.41, 5.76)
Cardiovascular	Tachycardia	4.69 (4.18, 5.21)	4.04 (3.3, 4.77)	5.32 (4.24, 6.51)	4.69 (3.91, 5.55)	5.75 (4.67, 6.92)	4.29 (3.71, 4.88)
Gastrointestinal	Diarrhea	4.68 (4.09, 5.27)	4.62 (3.7, 5.52)	4.42 (3.21, 5.68)	4.78 (3.82, 5.79)	5.15 (4, 6.33)	4.62 (3.88, 5.33)
Mental health	Mood disorder	4.57 (3.93, 5.21)	6.79 (5.27, 8.26)	4.06 (2.67, 5.5)	4.78 (3.73, 5.87)	5.58 (4.11, 7.09)	4.04 (3.33, 4.75)
Neurologic	Headache	3.80 (3.32, 4.28)	7.09 (5.79, 8.35)	5.6 (4.24, 6.97)	3 (2.19, 3.85)	5.24 (4.13, 6.39)	3.32 (2.78, 3.85)
Endocrine	Obesity	3.49 (3.06, 3.92)	5.57 (4.45, 6.63)	5.11 (3.94, 6.29)	2.67 (2.06, 3.29)	3.09 (2.22, 3.98)	3.78 (3.26, 4.29)
Dermatologic	Skin rash	3.30 (2.67, 3.89)	3.22 (2.32, 4.12)	3.29 (2, 4.61)	3.3 (2.28, 4.34)	2.37 (1.29, 3.47)	3.66 (2.87, 4.42)
Mental health	Substance abuse	3.24 (2.55, 3.92)	3.43 (2.12, 4.66)	4.22 (2.41, 6.05)	2.59 (1.58, 3.62)	5.28 (3.71, 6.89)	2.35 (1.59, 3.1)
Neurologic	Stroke	3.11 (2.6, 3.59)	0.83 (0.45, 1.24)	3.24 (2.24, 4.29)	5.44 (4.43, 6.5)	3.51 (2.51, 4.56)	2.94 (2.36, 3.51)
Cardiovascular	Bradycardia	2.63 (2.14, 3.1)	1.43 (0.97, 1.91)	2.26 (1.38, 3.22)	4.17 (3.24, 5.16)	2.03 (1.18, 2.91)	2.89 (2.28, 3.49)
Cardiovascular	Heart failure	2.02 (1.67, 2.37)	0.2 (0.01, 0.41)	1.72 (1.05, 2.46)	4.88 (4.08, 5.71)	2.07 (1.37, 2.82)	1.97 (1.56, 2.38)
Neurologic	Smell problems	1.34 (1.13, 1.56)	2.09 (1.67, 2.54)	1.15 (0.75, 1.63)	0.8 (0.54, 1.13)	0.88 (0.54, 1.3)	1.42 (1.17, 1.68)
Cardiovascular	Myocarditis	0.32 (0.23, 0.45)	0.22 (0.11, 0.38)	0.3 (0.15, 0.57)	0.28 (0.16, 0.49)	0.29 (0.14, 0.55)	0.3 (0.2, 0.46)
Dermatologic	Hair loss	0.22 (0.17, 0.29)	0.7 (0.48, 0.93)	0.27 (0.12, 0.47)	0.06 (0.03, 0.11)	0.1 (0.01, 0.23)	0.29 (0.21, 0.37)
Organ	Sequelae	Overall	Sex		Comorbidity		
			Male	Female	No comorbidities	1-3 comorbidities	>3 comorbidities
Pulmonary	Shortness of breath	28.8 (27.55, 29.97)	28.04 (26.74, 29.28)	33.55 (29.64, 37.41)	20.27 (18.96, 21.5)	29.79 (27.95, 31.63)	37.5 (33.46, 41.72)
Mental health	Sleep disorder	19.51 (18.05, 20.92)	20.31 (18.77, 21.82)	15.22 (10.43, 19.87)	16.73 (14.94, 18.39)	18.39 (16.13, 20.64)	25.51 (20.91, 30.28)
Endocrine	Hyperlipidemia	17.09 (15.28, 18.79)	17.78 (15.87, 19.59)	11.96 (6.26, 17.47)	19.99 (17.47, 22.32)	14.29 (11.47, 17.02)	8.89 (4.27, 13.65)

Cardiovascular	Chest pain	13.84 (12.86, 14.76)	13.49 (12.46, 14.46)	18.01 (14.65, 21.37)	11.16 (10.09, 12.21)	13.44 (12.03, 14.84)	12.75 (9.86, 15.82)
General	Fatigue	13.59 (12.66, 14.49)	14.3 (13.31, 15.27)	7.21 (4.89, 9.62)	6.96 (6.13, 7.76)	15.2 (13.83, 16.6)	33.42 (29.67, 37.33)
Gastrointestinal	GERD	13.25 (11.97, 14.49)	14.16 (12.8, 15.49)	6.67 (2.83, 10.43)	8.96 (7.42, 10.44)	13.71 (11.74, 15.69)	22.75 (18.73, 26.91)
Coagulation	Thromboembolism	11.35 (10.59, 12.12)	12.02 (11.2, 12.85)	5.02 (3.51, 6.72)	6.96 (6.2, 7.76)	12.62 (11.52, 13.78)	18.39 (15.99, 20.95)
Pulmonary	Cough	10.24 (9.46, 10.98)	10.04 (9.22, 10.82)	13.29 (10.66, 15.95)	8.57 (7.69, 9.43)	9.11 (8.02, 10.2)	9.77 (7.58, 12.14)
Musculoskeletal	Muscle weakness	9.81 (9.05, 10.55)	10.62 (9.81, 11.42)	2.85 (1.28, 4.56)	3.62 (3.04, 4.22)	12.2 (11.04, 13.4)	28.12 (24.86, 31.5)
Musculoskeletal	Joint pain	8.92 (7.67, 10.1)	9.65 (8.32, 10.92)	3.3 (-0.34, 6.93)	5.6 (4.22, 6.94)	9.65 (7.78, 11.5)	15.75 (11.5, 20.14)
Mental health	Anxiety	8.75 (7.96, 9.55)	9.2 (8.35, 10.06)	5.86 (3.3, 8.45)	4.84 (4.05, 5.61)	9.14 (7.99, 10.35)	18.4 (15.48, 21.45)
Endocrine	Diabetes mellitus	8.47 (7.46, 9.44)	9.18 (8.09, 10.25)	3.37 (1.29, 5.55)	8.11 (6.93, 9.24)	7.98 (6.18, 9.83)	10.59 (5.91, 15.81)
Cardiovascular	Arrythmias	7.85 (7.08, 8.58)	7.51 (6.69, 8.28)	10.9 (8.37, 13.49)	6.76 (5.91, 7.58)	6.82 (5.75, 7.91)	8.11 (5.89, 10.54)
Mental health	Depression	7.61 (6.8, 8.42)	7.83 (6.97, 8.69)	7.25 (4.1, 10.41)	4.98 (4.07, 5.86)	7.98 (6.75, 9.23)	12.77 (10.13, 15.58)
Kidney	Chronic kidney disease	7.19 (5.78, 8.55)	7.84 (6.33, 9.31)	3.34 (0.12, 6.85)	3.60 (2.42, 4.75)	10.57 (8.08, 13.08)	20.17 (13.92, 26.75)
Gastrointestinal	Constipation	7.13 (6.33, 7.9)	7.29 (6.44, 8.11)	6.01 (3.44, 8.64)	3.31 (2.6, 3.99)	8.44 (7.27, 9.65)	14.74 (11.76, 17.87)
Neurologic	Memory problems	6.46 (5.78, 7.13)	6.99 (6.25, 7.71)	2.05 (0.7, 3.54)	4.14 (3.48, 4.81)	6.44 (5.45, 7.46)	13.32 (10.85, 16.01)
Pulmonary	Hypoxemia	6.43 (5.94, 6.93)	6.89 (6.37, 7.44)	2.39 (1.58, 3.37)	3.03 (2.6, 3.49)	7.69 (6.97, 8.46)	15.81 (13.81, 17.92)
Cardiovascular	Acute coronary disease	6.18 (5.37, 6.96)	7.22 (6.31, 8.12)	0.18 (-0.83, 1.36)	2.69 (2.03, 3.33)	8.05 (6.72, 9.4)	17.39 (13.74, 21.19)
Kidney	Acute kidney injury	6.07 (5.46, 6.69)	6.92 (6.25, 7.61)	1.30 (0.36, 2.15)	2.62 (2.14, 3.14)	8.87 (7.91, 9.88)	25.07 (21.82, 28.42)
Cardiovascular	Tachycardia	4.69 (4.18, 5.21)	4.63 (4.09, 5.19)	5.31 (3.64, 7.12)	2.83 (2.37, 3.34)	5.02 (4.29, 5.81)	8.18 (6.49, 10.01)
Gastrointestinal	Diarrhea	4.68 (4.09, 5.27)	4.76 (4.13, 5.38)	4.62 (2.65, 6.68)	2.94 (2.39, 3.49)	4.44 (3.61, 5.3)	8.41 (6.28, 10.72)
Mental	Mood	4.57	4.71	4.39	3.87	4.14	7.1

health	disorder	(3.93, 5.21)	(4.04, 5.39)	(1.9, 6.91)	(3.01, 4.69)	(3.16, 5.14)	(5.17, 9.22)
Neurologic	Headache	3.8 (3.32, 4.28)	3.5 (2.98, 4.01)	11.44 (8.45, 14.46)	3.74 (3.09, 4.36)	3.45 (2.71, 4.21)	4.14 (2.64, 5.79)
Endocrine	Obesity	3.49 (3.06, 3.92)	3.54 (3.09, 3.99)	4.25 (2.17, 6.3)	3.54 (2.99, 4.07)	3.09 (2.43, 3.76)	3.55 (2.18, 4.99)
Dermatologic	Skin rash	3.3 (2.67, 3.89)	3.05 (2.39, 3.68)	5.77 (3.55, 8.04)	2.7 (1.99, 3.42)	3.39 (2.48, 4.29)	2.56 (0.8, 4.45)
Mental health	Substance abuse	3.24 (2.55, 3.92)	3.53 (2.8, 4.26)	0.92 (-0.78, 2.68)	3.49 (2.57, 4.38)	2.33 (1.31, 3.37)	4.64 (2.64, 6.79)
Neurologic	Stroke	3.11 (2.6, 3.59)	3.31 (2.76, 3.84)	1.46 (0.52, 2.58)	1.32 (0.9, 1.74)	3.69 (2.95, 4.46)	6.53 (4.67, 8.55)
Cardiovascular	Bradycardia	2.63 (2.14, 3.1)	2.73 (2.21, 3.24)	1.67 (0.67, 2.84)	1.84 (1.35, 2.33)	2.95 (2.28, 3.66)	3.27 (1.74, 4.99)
Cardiovascular	Heart failure	2.02 (1.67, 2.37)	2.27 (1.88, 2.66)	0.33 (-0.08, 0.9)	0.81 (0.57, 1.09)	2.8 (2.25, 3.39)	9.12 (7.22, 11.16)
Neurologic	Smell problems	1.34 (1.13, 1.56)	1.28 (1.06, 1.51)	1.7 (1.13, 2.39)	1.35 (1.07, 1.64)	1.1 (0.82, 1.42)	0.89 (0.46, 1.47)
Cardiovascular	Myocarditis	0.32 (0.23, 0.45)	0.34 (0.23, 0.48)	0 (0, 0.01)	0.11 (0.06, 0.19)	0.2 (0.11, 0.34)	0.57 (0.29, 1.08)
Dermatologic	Hair loss	0.22 (0.17, 0.29)	0.13 (0.08, 0.2)	3.59 (2.46, 4.84)	0.31 (0.21, 0.42)	0.2 (0.12, 0.3)	0.1 (0, 0.27)

Post-acute sequelae were ascertained from 30 days after infection until end of follow-up.
Estimates of burdens per 1000 COVID-19 patients at 6-months are presented.

Supplementary Table 6: Differences in burden of individual Post-acute Sequelae of COVID-19 by age, race, sex, and health status in the overall cohort

Organ	Sequelae	Age >70 compare to Age ≤60	Black compared to White	Male compared to Female	High comorbidity compared to low comorbidity
Kidney	Acute kidney injury	11.00 (9.66, 12.38)	4.06 (2.49, 5.73)	5.63 (4.45, 6.73)	22.50 (19.14, 25.95)
Mental health	Anxiety	7.31 (5.45, 9.21)	1.33 (-0.55, 3.26)	3.38 (0.58, 6.08)	13.55 (10.44, 16.79)
Cardiovascular	Bradycardia	2.73 (1.74, 3.81)	-0.87 (-1.86, 0.26)	1.07 (-0.18, 2.2)	1.45 (-0.15, 3.22)
Cardiovascular	Chest pain	-6.6 (-8.74, -4.54)	3.4 (0.96, 5.67)	-4.51 (-7.99, -0.91)	1.65 (-1.59, 4.79)
Kidney	Chronic kidney disease	18.36 (14.43, 22.21)	-0.89 (-3.71, 1.90)	4.5 (0.73, 8.13)	16.59 (10.10, 23.54)
Gastrointestinal	Constipation	9.76 (8.1, 11.52)	1.75 (-0.07, 3.7)	1.28 (-1.31, 4.02)	11.45 (8.44, 14.66)
Pulmonary	Cough	-1.71 (-3.28, -0.12)	3 (1.32, 4.82)	-3.25 (-5.9, -0.6)	1.2 (-1.07, 3.64)
Mental health	Depression	-0.66 (-2.6, 1.29)	0.56 (-1.27, 2.43)	0.6 (-2.56, 3.65)	7.82 (5.07, 10.67)
Gastrointestinal	Diarrhea	0.18 (-1.11, 1.41)	0.54 (-0.8, 1.92)	0.15 (-2.02, 2.07)	5.5 (3.32, 7.82)
Endocrine	Diabetes mellitus	6.41 (4.21, 8.55)	4.04 (1.43, 6.53)	5.78 (3.41, 8.19)	2.52 (-2.44, 7.74)
General	Fatigue	17.39 (15.43, 19.41)	0.63 (-1.44, 2.85)	7.1 (4.47, 9.55)	26.45 (22.7, 30.45)
Gastrointestinal	Gerd	9.49 (6.61, 12.27)	-3.01 (-5.91, -0.17)	7.56 (3.38, 11.59)	13.84 (9.56, 18.24)
Dermatologic	Hair loss	-0.63 (-0.87, -0.41)	-0.18 (-0.31, -0.03)	-3.46 (-4.72, -2.31)	-0.2 (-0.36, -0.02)
Neurologic	Headache	-4.09 (-5.6, -2.5)	1.92 (0.63, 3.2)	-7.94 (-10.88, -4.94)	0.4 (-1.28, 2.25)
Cardiovascular	Arrythmias	-1.24 (-2.95, 0.38)	-0.17 (-1.94, 1.55)	-3.37 (-6.13, -0.73)	1.42 (-1.17, 3.86)
Cardiovascular	Heart failure	4.67 (3.87, 5.51)	0.09 (-0.71, 0.95)	1.93 (1.28, 2.51)	8.3 (6.36, 10.39)
Endocrine	Hyperlipidemia	-8.5 (-12.92, -4.1)	-2.47 (-6.43, 1.44)	5.85 (0.16, 11.94)	-11.04 (-16.32, -5.93)
Pulmonary	Hypoxemia	7.53 (6.52, 8.6)	-0.91 (-1.98, 0.22)	4.5 (3.42, 5.48)	12.81 (10.74, 15.01)
Musculoskeletal	Joint pain	4.92 (2.23, 7.65)	1.76 (-1.1, 4.54)	6.38 (2.63, 10.21)	10.1 (5.76, 14.66)
Neurologic	Memory problems	12.75 (11.06, 14.44)	-0.96 (-2.34, 0.6)	4.92 (3.32, 6.54)	9.18 (6.67, 11.93)
Cardiovascular	Acute coronary disease	13.93 (11.87, 16.02)	-0.83 (-2.52, 0.94)	7.05 (5.53, 8.35)	14.66 (11.03, 18.55)
Mental health	Mood disorder	-1.98 (-3.82, -0.18)	1.56 (-0.05, 3.15)	0.34 (-2.24, 2.92)	3.29 (1.19, 5.51)
Musculoskeletal	Muscle weakness	16.96 (15.16, 18.69)	1.53 (-0.33, 3.39)	7.81 (5.75, 9.64)	24.47 (21, 28.02)
Cardiovascular	Myocarditis	0.07 (-0.14, 0.3)	-0.02 (-0.23, 0.27)	0.34 (0.23, 0.48)	0.46 (0.17, 0.97)

Endocrine	Obesity	-2.88 (-4.16, -1.68)	-0.69 (-1.69, 0.26)	-0.67 (-2.86, 1.38)	0.01 (-1.49, 1.55)
Pulmonary	Shortness of breath	4.28 (1.67, 6.92)	1.74 (-1.16, 4.71)	-5.46 (-9.47, -1.41)	17.26 (13.1, 21.53)
Dermatologic	Skin rash	0.1 (-1.27, 1.54)	-1.29 (-2.63, 0.08)	-2.7 (-5.1, -0.41)	-0.14 (-2.06, 1.9)
Mental health	Sleep disorder	-5.59 (-9.45, -2)	-3.22 (-6.5, 0.17)	5.19 (0.11, 9.96)	8.77 (3.69, 14.03)
Neurologic	Smell problems	-1.27 (-1.8, -0.75)	-0.54 (-0.97, -0.06)	-0.41 (-1.14, 0.19)	-0.45 (-0.99, 0.18)
Neurologic	Stroke	4.61 (3.53, 5.71)	0.57 (-0.6, 1.76)	1.83 (0.59, 2.94)	5.21 (3.28, 7.25)
Mental health	Substance abuse	-0.83 (-2.34, 0.76)	2.92 (1.26, 4.73)	2.61 (0.82, 4.47)	1.13 (-1, 3.49)
Cardiovascular	Tachycardia	0.68 (-0.51, 1.8)	1.46 (0.17, 2.77)	-0.68 (-2.5, 1.03)	5.34 (3.47, 7.31)
Coagulation	Thromboembolism	10.04 (8.34, 11.68)	2.71 (0.82, 4.56)	7.01 (4.97, 8.67)	11.46 (8.88, 14.2)
Differences in burden per 1000 COVID-19 patients and 95% confidence intervals at 6-months are presented.					

Supplementary Table 7: Burden of Post-acute Sequelae of COVID-19 individual sequelae in the overall cohort and by age, race, sex, and health status in non-hospitalized COVID-19

Organ	Sequelae	Overall	Age			Race	
			Age ≤ 60	Age >60 - ≤ 70	Age >70	Black	White
Pulmonary	Shortness of breath	23.29 (21.99, 24.52)	21.28 (19.64, 22.85)	25.41 (22.63, 28.1)	18.31 (16.22, 20.32)	23.37 (20.8, 25.86)	23.35 (21.77, 24.85)
Mental health	Sleep disorder	14.16 (12.62, 15.6)	22.09 (19.03, 24.98)	13.78 (10.17, 17.28)	11.26 (8.8, 13.63)	12.64 (9.63, 15.52)	14.64 (12.82, 16.35)
Endocrine	Hyperlipidemia	16.18 (14.23, 18.01)	21.46 (17.87, 24.85)	19.94 (15.11, 24.53)	11.89 (8.8, 14.83)	14.49 (10.89, 17.92)	16.68 (14.28, 18.93)
Cardiovascular	Chest pain	11.38 (10.34, 12.36)	14.72 (13.08, 16.3)	13.33 (10.9, 15.71)	6.66 (5.1, 8.19)	12.46 (10.26, 14.61)	11.08 (9.9, 12.22)
General	Fatigue	7.79 (6.85, 8.7)	4.18 (3.24, 5.12)	6.83 (4.97, 8.69)	10.77 (8.98, 12.51)	7.19 (5.33, 9.03)	7.9 (6.77, 8.98)
Gastrointestinal	GERD	7.9 (6.56, 9.17)	7.21 (5.39, 8.95)	8.85 (5.95, 11.66)	7.17 (4.86, 9.4)	5.75 (3.27, 8.17)	8.61 (6.95, 10.19)
Coagulation	Thromboembolism	7.36 (6.6, 8.09)	3.88 (3.1, 4.68)	7.49 (5.96, 9.06)	9.71 (8.35, 11.06)	8.12 (6.54, 9.73)	7.06 (6.19, 7.91)
Pulmonary	Cough	9.11 (8.29, 9.91)	9.26 (8.08, 10.42)	12.35 (10.43, 14.27)	6.22 (4.93, 7.5)	10.92 (9.19, 12.66)	8.45 (7.49, 9.38)
Musculoskeletal	Muscle weakness	5.57 (4.8, 6.32)	1.72 (1.08, 2.37)	3.66 (2.22, 5.12)	10.32 (8.7, 11.89)	5.77 (4.18, 7.36)	5.52 (4.61, 6.4)
Musculoskeletal	Joint pain	4.38 (3.06, 5.61)	4.39 (2.52, 6.18)	4.82 (1.85, 7.7)	3.99 (1.77, 6.14)	5.38 (2.59, 8.08)	4.02 (2.51, 5.47)
Mental health	Anxiety	4.14 (3.34, 4.9)	3.25 (2.04, 4.41)	5.83 (3.89, 7.76)	5.12 (3.72, 6.51)	4.24 (2.59, 5.87)	4.3 (3.37, 5.19)
Endocrine	Diabetes mellitus	5.51 (4.47, 6.5)	3.26 (2.15, 4.34)	6.51 (4.19, 8.82)	6.34 (4.38, 8.25)	7.31 (4.93, 9.65)	4.7 (3.57, 5.79)
Cardiovascular	Arrythmias	6.37 (5.55, 7.16)	7.38 (6.3, 8.44)	5.12 (3.51, 6.74)	4.26 (2.92, 5.59)	5.99 (4.4, 7.57)	6.5 (5.53, 7.45)
Mental health	Depression	3.95 (3.11, 4.74)	6.63 (5.01, 8.19)	3.4 (1.44, 5.35)	2.56 (1.24, 3.86)	3.68 (1.93, 5.39)	3.92 (2.97, 4.84)
Kidney	Chronic kidney disease	1.66 (0.19, 3.08)	0.43 (-0.28, 1.15)	0.92 (-1.68, 3.54)	2.13 (-1.83, 5.99)	1.11 (-0.84, 3.06)	2.11 (0.03, 4.13)
Gastrointestinal	Constipation	3.91 (3.07, 4.71)	1.89 (1.05, 2.73)	2.2 (0.56, 3.85)	6.57 (4.96, 8.14)	4.6 (2.8, 6.38)	3.55 (2.59, 4.48)
Neurologic	Memory problems	3.51 (2.81, 4.19)	1.34 (0.78, 1.92)	1.25 (0.07, 2.47)	7.28 (5.66, 8.88)	2.79 (1.56, 4.06)	4.02 (3.14, 4.88)
Pulmonary	Hypoxemia	3.82	1.81	3.85	5.31	3.05	4.31

		(3.35, 4.29)	(1.37, 2.28)	(2.94, 4.8)	(4.41, 6.21)	(2.22, 3.95)	(3.7, 4.92)
Cardiovascular	Acute coronary disease	2.55 (1.71, 3.35)	0.46 (-0.14, 1.06)	1.73 (-0.03, 3.49)	5.79 (3.74, 7.77)	1.58 (0.1, 3.07)	3.04 (1.93, 4.11)
Kidney	Acute kidney injury	1.28 (0.68, 1.86)	0.38 (-0.14, 0.92)	0.88 (-0.31, 2.11)	2.29 (1.12, 3.44)	1.47 (0.09, 2.88)	1.02 (0.36, 1.66)
Cardiovascular	Tachycardia	2.57 (2.06, 3.07)	2.87 (2.14, 3.61)	2.67 (1.57, 3.83)	1.88 (1.08, 2.71)	2.31 (1.25, 3.42)	2.58 (2, 3.17)
Gastrointestinal	Diarrhea	2.98 (2.35, 3.58)	3.89 (2.96, 4.82)	2.2 (0.94, 3.49)	2.16 (1.14, 3.19)	3.66 (2.41, 4.93)	2.92 (2.15, 3.66)
Mental health	Mood disorder	2.94 (2.27, 3.58)	5.86 (4.3, 7.36)	2.09 (0.62, 3.58)	2.56 (1.44, 3.68)	3.94 (2.37, 5.49)	2.49 (1.76, 3.19)
Neurologic	Headache	3.06 (2.55, 3.55)	6.27 (4.93, 7.56)	4.84 (3.36, 6.32)	2.23 (1.34, 3.15)	4.46 (3.27, 5.64)	2.58 (2.02, 3.12)
Endocrine	Obesity	2.22 (1.76, 2.64)	4.56 (3.41, 5.66)	3.22 (1.98, 4.43)	1.12 (0.49, 1.75)	1.19 (0.29, 2.08)	2.68 (2.14, 3.2)
Dermatologic	Skin rash	2.64 (1.96, 3.29)	2.7 (1.77, 3.62)	2.47 (1.06, 3.9)	2.59 (1.44, 3.74)	1.79 (0.61, 2.99)	2.95 (2.11, 3.76)
Mental health	Substance abuse	0.98 (0.27, 1.65)	2.14 (0.82, 3.41)	0.34 (-1.54, 2.19)	0.08 (-0.98, 1.14)	1.85 (0.21, 3.46)	0.49 (-0.29, 1.23)
Neurologic	Stroke	2.08 (1.55, 2.61)	0.51 (0.13, 0.92)	1.93 (0.88, 3.03)	3.6 (2.5, 4.7)	1.8 (0.73, 2.91)	2.09 (1.48, 2.71)
Cardiovascular	Bradycardia	1.6 (1.09, 2.11)	1.2 (0.73, 1.7)	1.24 (0.32, 2.24)	1.82 (0.84, 2.82)	1.19 (0.27, 2.17)	1.77 (1.13, 2.41)
Cardiovascular	Heart failure	0.75 (0.38, 1.11)	0.02 (-0.17, 0.24)	0.32 (-0.35, 1.05)	1.83 (1.01, 2.66)	0.4 (-0.32, 1.18)	0.81 (0.39, 1.23)
Neurologic	Smell problems	1.25 (1.03, 1.47)	2.01 (1.59, 2.46)	1.04 (0.63, 1.55)	0.63 (0.35, 0.97)	0.78 (0.42, 1.22)	1.3 (1.04, 1.57)
Cardiovascular	Myocarditis	0.17 (0.1, 0.26)	0.16 (0.07, 0.31)	0.11 (0.03, 0.29)	0.12 (0.04, 0.27)	0.12 (0.03, 0.32)	0.17 (0.09, 0.29)
Dermatologic	Hair loss	0.19 (0.13, 0.25)	0.59 (0.38, 0.83)	0.26 (0.1, 0.46)	0.06 (0.02, 0.11)	0.03 (-0.07, 0.14)	0.26 (0.19, 0.33)
Organ	Sequelae	Overall	Sex		Comorbidity		
			Male	Female	No comorbidities	1-3 comorbidities	>3 comorbidities
Pulmonary	Shortness of breath	23.29 (21.99, 24.52)	22.19 (20.82, 23.48)	30.24 (26.3, 34.16)	17.64 (16.33, 18.9)	21.79 (19.92, 23.59)	18.72 (14.25, 23.09)
Mental health	Sleep disorder	14.16 (12.62, 15.6)	14.44 (12.81, 15.98)	13.72 (8.82, 18.51)	13.85 (12.04, 15.57)	12.21 (9.82, 14.49)	10.51 (5.3, 15.62)
Endocrine	Hyperlipidemia	16.18 (14.23, 18.01)	17.01 (14.93, 18.95)	10.47 (4.61, 16.14)	19.18 (16.58, 21.62)	12.17 (9.11, 15.06)	6.9 (1.26, 12.37)

Cardiovascular	Chest pain	11.38 (10.34, 12.36)	10.94 (9.85, 11.98)	16.14 (12.71, 19.56)	9.9 (8.81, 10.95)	9.65 (8.18, 11.08)	7.22 (3.83, 10.59)
General	Fatigue	7.79 (6.85, 8.7)	8.01 (6.99, 8.97)	5.48 (3.12, 7.95)	4.93 (4.12, 5.72)	7.19 (5.87, 8.49)	9.86 (5.87, 13.81)
Gastrointestinal	GERD	7.9 (6.56, 9.17)	8.37 (6.94, 9.72)	4.91 (0.99, 8.81)	7.26 (5.7, 8.76)	7.35 (5.3, 9.31)	5.49 (1.08, 9.85)
Coagulation	Thromboembolism	7.36 (6.6, 8.09)	7.71 (6.9, 8.49)	3.42 (1.97, 5.09)	4.65 (3.94, 5.37)	7.05 (6, 8.09)	8.7 (6.17, 11.3)
Pulmonary	Cough	9.11 (8.29, 9.91)	8.9 (8.02, 9.75)	12.25 (9.56, 15)	7.91 (7.02, 8.78)	7.43 (6.27, 8.56)	6.73 (4.17, 9.37)
Musculoskeletal	Muscle weakness	5.57 (4.8, 6.32)	5.98 (5.15, 6.79)	1.69 (0.11, 3.45)	2.01 (1.46, 2.58)	6.45 (5.32, 7.56)	9.59 (6.13, 13.05)
Musculoskeletal	Joint pain	4.38 (3.06, 5.61)	4.83 (3.43, 6.16)	1.08 (-2.63, 4.76)	3.46 (2.06, 4.8)	4.55 (2.59, 6.43)	1.62 (-3.17, 6.33)
Mental health	Anxiety	4.14 (3.34, 4.9)	4.34 (3.48, 5.16)	3.54 (0.97, 6.14)	2.73 (1.98, 3.47)	3.88 (2.73, 5)	4.79 (1.64, 7.96)
Endocrine	Diabetes mellitus	5.51 (4.47, 6.5)	5.87 (4.75, 6.94)	2.44 (0.33, 4.65)	5.73 (4.54, 6.87)	4.54 (2.66, 6.38)	2.93 (-2.35, 8.68)
Cardiovascular	Arrythmias	6.37 (5.55, 7.16)	5.9 (5.04, 6.74)	10.24 (7.64, 12.9)	6.16 (5.29, 7.01)	4.78 (3.65, 5.88)	2.78 (0.22, 5.39)
Mental health	Depression	3.95 (3.11, 4.74)	4.03 (3.14, 4.89)	4.86 (1.67, 8.04)	3.28 (2.37, 4.17)	3.88 (2.63, 5.1)	2.44 (-0.44, 5.35)
Kidney	Chronic kidney disease	1.66 (0.19, 3.08)	1.55 (-0.03, 3.06)	2.86 (-0.49, 6.57)	1.82 (0.63, 3.01)	1.61 (-0.92, 4.08)	1.66 (-1.75, 5.03)
Gastrointestinal	Constipation	3.91 (3.07, 4.71)	3.81 (2.92, 4.68)	4.83 (2.19, 7.53)	2.08 (1.38, 2.78)	4.09 (2.9, 5.26)	4.28 (0.92, 7.64)
Neurologic	Memory problems	3.51 (2.81, 4.19)	3.76 (3.01, 4.5)	1.07 (-0.28, 2.59)	2.6 (1.95, 3.26)	2.44 (1.47, 3.4)	5.45 (2.67, 8.28)
Pulmonary	Hypoxemia	3.82 (3.35, 4.29)	4.02 (3.52, 4.52)	1.67 (0.89, 2.64)	1.87 (1.5, 2.26)	3.67 (3.03, 4.33)	6.87 (4.89, 8.94)
Cardiovascular	Acute coronary disease	2.55 (1.71, 3.35)	2.94 (2, 3.84)	-0.14 (-1.18, 1.08)	1.43 (0.78, 2.09)	2.39 (1.07, 3.68)	4.55 (0.58, 8.51)
Kidney	Acute kidney injury	1.28 (0.68, 1.86)	1.47 (0.82, 2.11)	0.31 (-0.69, 1.11)	0.86 (0.43, 1.31)	1.51 (0.63, 2.39)	0.72 (-2.55, 3.99)
Cardiovascular	Tachycardia	2.57 (2.06, 3.07)	2.4 (1.87, 2.93)	4.1 (2.44, 5.91)	1.61 (1.18, 2.06)	2.49 (1.77, 3.22)	2.11 (0.35, 3.98)
Gastrointestinal	Diarrhea	2.98 (2.35, 3.58)	2.95 (2.28, 3.6)	3.72 (1.71, 5.85)	2.36 (1.81, 2.91)	2.25 (1.41, 3.08)	2.05 (-0.34, 4.51)
Mental	Mood	2.94	2.97	3.67	3.09	2.25	3.7

health	disorder	(2.27, 3.58)	(2.26, 3.65)	(1.1, 6.26)	(2.23, 3.93)	(1.22, 3.26)	(1.46, 5.99)
Neurologic	Headache	3.06<br>(2.55, 3.55)	2.78<br>(2.24, 3.31)	10.15<br>(7.09, 13.19)	3.34<br>(2.67, 3.98)	2.53<br>(1.74, 3.3)	2.79<br>(1.01, 4.65)
Endocrine	Obesity	2.22<br>(1.76, 2.64)	2.15<br>(1.68, 2.6)	3.89<br>(1.74, 6.01)	2.75<br>(2.19, 3.28)	1.65<br>(0.96, 2.31)	-0.13<br>(-1.65, 1.4)
Dermatologic	Skin rash	2.64<br>(1.96, 3.29)	2.38<br>(1.66, 3.07)	5.09<br>(2.81, 7.44)	2.27<br>(1.54, 2.99)	2.4<br>(1.44, 3.36)	1.63<br>(-0.49, 3.83)
Mental health	Substance abuse	0.98<br>(0.27, 1.65)	1.13<br>(0.37, 1.85)	-0.13<br>(-1.85, 1.64)	1.69<br>(0.78, 2.56)	0.17<br>(-0.89, 1.2)	0.18<br>(-2.09, 2.5)
Neurologic	Stroke	2.08<br>(1.55, 2.61)	2.14<br>(1.56, 2.71)	1.36<br>(0.39, 2.54)	0.75<br>(0.35, 1.18)	2.38<br>(1.61, 3.15)	3.26<br>(1.13, 5.48)
Cardiovascular	Bradycardia	1.6<br>(1.09, 2.11)	1.58<br>(1.04, 2.12)	1.43<br>(0.42, 2.65)	1.38<br>(0.9, 1.89)	1.45<br>(0.77, 2.15)	0.35<br>(-1.42, 2.22)
Cardiovascular	Heart failure	0.75<br>(0.38, 1.11)	0.81<br>(0.41, 1.21)	0.21<br>(-0.21, 0.81)	0.43<br>(0.2, 0.69)	0.84<br>(0.3, 1.39)	1.09<br>(-0.93, 3.19)
Neurologic	Smell problems	1.25<br>(1.03, 1.47)	1.19<br>(0.96, 1.44)	1.56<br>(1, 2.26)	1.27<br>(1, 1.57)	0.96<br>(0.68, 1.29)	0.49<br>(0.06, 1.1)
Cardiovascular	Myocarditis	0.17<br>(0.1, 0.26)	0.17<br>(0.1, 0.28)	0<br>(0, 0.01)	0.06<br>(0.02, 0.12)	0.09<br>(0.03, 0.19)	0.21<br>(0.06, 0.54)
Dermatologic	Hair loss	0.19<br>(0.13, 0.25)	0.11<br>(0.06, 0.18)	3.12<br>(1.99, 4.37)	0.28<br>(0.18, 0.38)	0.14<br>(0.05, 0.23)	0.12<br>(0.01, 0.31)

Post-acute sequelae were ascertained from 30 days after infection until end of follow-up.
Estimates of burdens per 1000 COVID-19 patients at 6-months are presented.

Supplementary Table 8: Burden of Post-acute Sequelae of COVID-19 individual sequelae in the overall cohort and by age, race, sex, and health status in hospitalized COVID-19

Organ	Sequelae	Overall	Age			Race	
			Age ≤ 60	Age >60 - ≤ 70	Age >70	Black	White
Pulmonary	Shortness of breath	56.32 (52.5, 60.08)	45.58 (39.3, 52.12)	65.22 (57.23, 73.39)	57.18 (51.57, 62.79)	53.82 (47.43, 60.33)	58.11 (53.15, 63.04)
Mental health	Sleep disorder	47.37 (42.35, 52.31)	54.14 (42, 66.64)	48.22 (37.95, 58.8)	47.3 (40.65, 53.95)	32.66 (24.68, 40.82)	56.77 (50.12, 63.38)
Endocrine	Hyperlipide mia	16.72 (11.28, 22.12)	18.22 (5.56, 31.23)	22.54 (10.45, 35.01)	13.55 (6.69, 20.54)	14.83 (6.24, 23.63)	17.05 (9.93, 24.18)
Cardiovasc ular	Chest pain	29.6 (26.52, 32.66)	38.65 (31.96, 45.64)	32.1 (25.6, 38.88)	23.59 (19.75, 27.5)	35.52 (29.58, 41.68)	27.6 (23.87, 31.37)
General	Fatigue	43.64 (40.58, 46.66)	14.05 (10.62, 17.82)	35.91 (30.45, 41.61)	66.68 (61.23, 72.1)	36.61 (31.68, 41.66)	47.57 (43.55, 51.59)
Gastrointest inal	Gerd	43 (38.45, 47.49)	21.06 (14, 28.58)	39.91 (31.23, 48.92)	55.57 (48.59, 62.55)	30.46 (23.7, 37.41)	51.35 (45.12, 57.57)
Coagulation	Thromboem bolism	28.18 (25.68, 30.69)	21.91 (17.94, 26.28)	31.28 (26.21, 36.71)	30.09 (26.37, 33.93)	30.44 (26.03, 35.09)	27.44 (24.31, 30.64)
Pulmonary	Cough	15.73 (13.52, 17.98)	13.38 (9.38, 17.87)	18.3 (13.79, 23.26)	14.74 (11.78, 17.86)	14.97 (11.27, 19.01)	15.94 (13.14, 18.84)
Musculoskel etal	Muscle weakness	31.2 (28.78, 33.61)	8.81 (6.46, 11.49)	29.35 (24.65, 34.3)	49.93 (45.36, 54.5)	29.43 (25.21, 33.81)	31.98 (28.92, 35.06)
Musculoskel etal	Joint pain	35.78 (31.49, 40)	33.61 (25.57, 42.02)	34.5 (25.89, 43.43)	36.98 (31.01, 43)	29.11 (21.82, 36.57)	39.16 (33.69, 44.65)
Mental health	Anxiety	28.15 (25.33, 30.95)	21.1 (15.65, 27)	28.24 (22.44, 34.37)	30.97 (27.14, 34.9)	25.17 (20.35, 30.26)	30.24 (26.65, 33.86)
Endocrine	Diabetes mellitus	26.9 (23.02, 30.85)	21.39 (15.93, 27.38)	33.47 (25.06, 42.57)	25.74 (19.86, 31.95)	28.99 (21.81, 36.6)	26.3 (21.55, 31.27)
Cardiovasc ular	Arrythmias	12.92 (10.79, 15.08)	13.16 (9.4, 17.34)	11.79 (7.82, 16.18)	12.9 (9.84, 16.1)	12.18 (8.71, 15.94)	12.84 (10.14, 15.64)
Mental health	Depression	29.33 (26.26, 32.37)	33.45 (26.23, 41.06)	33.09 (26.67, 39.81)	27.05 (23.14, 31.06)	28.03 (22.52, 33.75)	30.29 (26.47, 34.15)
Kidney	Chronic kidney disease	36.41 (31.71, 41.11)	4.73 (2.42, 7.39)	31.97 (23.86, 40.66)	76.89 (65.12, 88.79)	26.93 (21.31, 32.81)	41.43 (34.49, 48.52)
Gastrointest inal	Constipation	23.96 (21.47, 26.43)	9.89 (6.74, 13.38)	23.62 (18.81, 28.69)	32 (27.95, 36.12)	24.85 (20.26, 29.66)	24.22 (21.13, 27.34)
Neurologic	Memory problems	22.62 (20.3, 24.93)	6.36 (4.16, 8.95)	16.83 (13.03, 20.97)	42.01 (37.04, 47.05)	17.74 (14.46, 21.24)	24.89 (21.76, 28.07)
Pulmonary	Hypoxemia	18.17	11.15	17.26	24.81	15.8	19.84

		(16.49, 19.87)	(8.72, 14.01)	(14.09, 20.75)	(21.9, 27.82)	(13.01, 18.87)	(17.6, 22.14)
Cardiovascular	Acute coronary disease	26.19 (23.31, 29.07)	8.12 (5.6, 10.99)	31.25 (25.03, 37.81)	45.65 (39.21, 52.17)	20.89 (16.84, 25.18)	29.07 (24.99, 33.21)
Kidney	Acute kidney injury	28.11 (25.94, 30.26)	8.85 (6.73, 11.23)	26.55 (22.34, 31.00)	42.92 (38.95, 46.90)	34.41 (29.96, 38.99)	26.83 (24.22, 29.47)
Cardiovascular	Tachycardia	13.72 (12.05, 15.44)	14.4 (11.21, 18)	13.47 (10.32, 16.98)	12.83 (10.57, 15.24)	16.28 (13.04, 19.81)	12.84 (10.84, 14.97)
Gastrointestinal	Diarrhea	12.94 (11.08, 14.83)	12.37 (8.8, 16.36)	13.73 (10.18, 17.62)	12.29 (9.78, 14.92)	9.84 (7.07, 12.88)	14.16 (11.71, 16.7)
Mental health	Mood disorder	13.23 (10.97, 15.53)	17.97 (11.65, 24.87)	13.02 (8.86, 17.59)	12.1 (9.24, 15.1)	11.31 (7.11, 15.82)	13.63 (10.88, 16.49)
Neurologic	Headache	7.47 (5.85, 9.14)	16.05 (10.76, 21.82)	8.17 (4.67, 12.11)	5.28 (3.35, 7.4)	7.53 (4.43, 10.94)	7.67 (5.71, 9.76)
Endocrine	Obesity	9.19 (7.67, 10.71)	15.41 (10.91, 20.18)	13.84 (10.06, 17.86)	6.41 (4.67, 8.24)	10.52 (7.74, 13.44)	8.91 (7.01, 10.87)
Dermatologic	Skin rash	6.8 (5.01, 8.65)	8.82 (5.31, 12.81)	7.54 (4.07, 11.46)	5.25 (2.88, 7.81)	5.07 (2.36, 8.13)	7.95 (5.58, 10.46)
Mental health	Substance abuse	16.04 (13.42, 18.7)	18.06 (11.78, 24.94)	26.57 (20.1, 33.45)	10.84 (7.94, 13.9)	20.12 (15.01, 25.5)	14.44 (11.31, 17.72)
Neurologic	Stroke	7.37 (6.02, 8.77)	4.3 (2.8, 6.14)	8.11 (5.4, 11.21)	9.32 (6.92, 11.87)	7.45 (5.11, 10.07)	7.61 (5.91, 9.41)
Cardiovascular	Bradycardia	6.71 (5.36, 8.1)	2.86 (1.32, 4.9)	5.83 (3.4, 8.72)	10.05 (7.65, 12.62)	3.85 (2.03, 5.98)	8.3 (6.45, 10.28)
Cardiovascular	Heart failure	8.17 (7.11, 9.26)	1.35 (0.73, 2.18)	7.57 (5.5, 9.92)	14.64 (12.42, 16.95)	8.05 (6.16, 10.11)	8.54 (7.2, 9.94)
Neurologic	Smell problems	1.5 (0.98, 2.16)	2.76 (1.49, 4.65)	0.86 (0.19, 2.11)	1.16 (0.55, 2.05)	1.02 (0.31, 2.17)	1.84 (1.14, 2.76)
Cardiovascular	Myocarditis	1 (0.61, 1.6)	0.48 (0.15, 1.3)	0.84 (0.32, 2.05)	0.84 (0.41, 1.66)	0.73 (0.29, 1.74)	0.97 (0.52, 1.74)
Dermatologic	Hair loss	0.43 (0.23, 0.7)	2.03 (1.04, 3.43)	0.14 (-0.11, 0.77)	0.05 (0, 0.18)	0.41 (0.07, 0.97)	0.48 (0.22, 0.87)

Organ (Level 1)	Sequelae (Level 2)	Overall	Sex		Comorbidity		
			Male	Female	No comorbidities	1-3 comorbidities	>3 comorbidities
Pulmonary	Shortness of breath	56.32 (52.5, 60.08)	54.88 (50.97, 58.74)	75.8 (60.15, 93.15)	47.64 (41.39, 54.18)	64.55 (58.46, 70.66)	71.44 (61.79, 81.21)
Mental health	Sleep disorder	47.37 (42.35, 52.31)	48.79 (43.6, 53.9)	28.29 (10.18, 49.33)	44.1 (35.65, 52.9)	46.44 (39.15, 53.76)	53.57 (42.7, 64.68)
Endocrine	Hyperlipidemia	16.72 (11.28, 22.12)	15.82 (10.24, 21.37)	30.72 (8.59, 55.41)	19.91 (10.09, 30.02)	19.49 (11.2, 27.92)	10.26 (0.47, 20.29)

Cardiovascular	Chest pain	29.6 (26.52, 32.66)	28.56 (25.42, 31.67)	46.01 (32.16, 61.87)	28.49 (23.31, 34.01)	33.06 (28.4, 37.8)	27.06 (20.45, 33.97)
General	Fatigue	43.64 (40.58, 46.66)	44.63 (41.44, 47.78)	25.84 (16.75, 36.7)	27.75 (23.24, 32.57)	47.46 (42.63, 52.34)	84.53 (74.98, 94.11)
Gastrointestinal	GERD	43 (38.45, 47.49)	44.4 (39.67, 49.07)	23.14 (8.33, 40.75)	30.76 (23.56, 38.4)	41.8 (35.21, 48.47)	56.43 (46.41, 66.69)
Coagulation	Thromboembolism	28.18 (25.68, 30.69)	28.37 (25.77, 30.99)	25.68 (17.88, 35.44)	31.08 (26.16, 36.43)	32.44 (28.39, 36.64)	30.84 (25.43, 36.56)
Pulmonary	Cough	15.73 (13.52, 17.98)	15.33 (13.07, 17.64)	21.61 (12.43, 33.13)	15.99 (12.14, 20.34)	16.66 (13.38, 20.13)	14.54 (10.02, 19.46)
Musculoskeletal	Muscle weakness	31.2 (28.78, 33.61)	32.32 (29.76, 34.84)	15.12 (9.06, 22.74)	20.55 (17, 24.45)	34.85 (30.88, 38.9)	64.82 (56.78, 72.95)
Musculoskeletal	Joint pain	35.78 (31.49, 40)	35.83 (31.41, 40.19)	35.36 (19.29, 53.97)	33.78 (26.74, 41.2)	32.95 (26.89, 39.1)	45.29 (35.03, 55.71)
Mental health	Anxiety	28.15 (25.33, 30.95)	27.99 (25.1, 30.86)	30.73 (18.58, 45.29)	26.22 (21.39, 31.42)	27.22 (23.15, 31.41)	35.75 (29.24, 42.5)
Endocrine	Diabetes mellitus	26.9 (23.02, 30.85)	27.9 (23.78, 32.1)	17.17 (7.81, 29.52)	34.65 (28.56, 41.09)	20.72 (15.22, 26.57)	20.14 (10.05, 31.82)
Cardiovascular	Arrythmias	12.92 (10.79, 15.08)	12.61 (10.43, 14.82)	16.77 (8.32, 27.47)	11.34 (7.92, 15.26)	12.8 (9.68, 16.09)	16.03 (11.13, 21.28)
Mental health	Depression	29.33 (26.26, 32.37)	29.08 (25.95, 32.19)	34.37 (20.28, 50.94)	27.05 (21.75, 32.75)	27.2 (22.89, 31.61)	36.83 (30.1, 43.83)
Kidney	Chronic kidney disease	36.41 (31.71, 41.11)	39.33 (34.29, 44.37)	2.23 (-5.06, 13.55)	21.72 (16.21, 27.88)	45.66 (37.54, 53.98)	65.25 (49.21, 81.95)
Gastrointestinal	Constipation	23.96 (21.47, 26.43)	24.19 (21.64, 26.75)	18.58 (9.26, 29.96)	17.2 (13.53, 21.22)	25.63 (21.84, 29.53)	37.1 (30.28, 44.13)
Neurologic	Memory problems	22.62 (20.3, 24.93)	23.56 (21.1, 26.01)	10.99 (5.68, 18.12)	22.43 (18.52, 26.66)	24.57 (20.96, 28.3)	27.76 (21.98, 33.88)
Pulmonary	Hypoxemia	18.17 (16.49, 19.87)	18.86 (17.07, 20.67)	10.02 (6.38, 14.98)	14.11 (11.26, 17.41)	23.28 (20.31, 26.4)	30.19 (25.36, 35.33)
Cardiovascular	Acute coronary disease	26.19 (23.31, 29.07)	29.21 (25.98, 32.42)	3.74 (0.52, 8.51)	15.4 (11.89, 19.3)	32.5 (27.65, 37.47)	42.77 (33.51, 52.36)
Kidney	Acute kidney injury	28.11 (25.94, 30.26)	30.22 (27.88, 32.56)	6.03 (2.94, 10.27)	19.97 (16.58, 23.71)	34.44 (30.61, 38.36)	69.87 (61.21, 78.66)
Cardiovascular	Tachycardia	13.72 (12.05, 15.44)	13.42 (11.71, 15.17)	17.01 (10.36, 25.68)	13.76 (10.83, 17.11)	12.61 (10.21, 15.22)	19.69 (15.54, 24.16)
Gastrointestinal	Diarrhea	12.94 (11.08, 14.83)	12.79 (10.89, 14.73)	14.47 (7.27, 23.65)	9.41 (6.7, 12.55)	12.89 (10.18, 15.78)	19.74 (14.84, 24.96)
Mental	Mood	13.23	13.61	8.15	13.63	13.94	11.06

health	disorder	(10.97, 15.53)	(11.29, 15.98)	(-1.05, 20.16)	(9.46, 18.24)	(10.67, 17.38)	(6.93, 15.57)
Neurologic	Headache	7.47<br>(5.85, 9.14)	6.59<br>(4.98, 8.26)	28.62<br>(15.98, 43.62)	7.06<br>(4.21, 10.31)	7.61<br>(5.32, 10.08)	7.05<br>(3.86, 10.64)
Endocrine	Obesity	9.19<br>(7.67, 10.71)	9.44<br>(7.89, 11.01)	6.03<br>(-0.88, 14.4)	11.85<br>(9.05, 14.89)	8.09<br>(5.91, 10.37)	9.5<br>(6.13, 13.06)
Dermatologic	Skin rash	6.8<br>(5.01, 8.65)	6.53<br>(4.71, 8.42)	10.67<br>(3.22, 20.44)	8.57<br>(5.37, 12.31)	7.87<br>(5.23, 10.73)	3.64<br>(0.24, 7.42)
Mental health	Substance abuse	16.04<br>(13.42, 18.7)	16.19<br>(13.46, 18.96)	13.07<br>(5.03, 23.49)	29.45<br>(23.5, 35.82)	11.88<br>(8.4, 15.56)	11.98<br>(7.29, 17.01)
Neurologic	Stroke	7.37<br>(6.02, 8.77)	7.8<br>(6.36, 9.3)	2.5<br>(0.22, 6.34)	6.88<br>(4.78, 9.44)	8.65<br>(6.47, 11.04)	10.02<br>(6.31, 14.11)
Cardiovascular	Bradycardia	6.71<br>(5.36, 8.1)	6.93<br>(5.51, 8.41)	3.83<br>(0.86, 8.8)	5.62<br>(3.49, 8.31)	8.21<br>(6.12, 10.5)	7.29<br>(4.07, 10.88)
Cardiovascular	Heart failure	8.17<br>(7.11, 9.26)	8.86<br>(7.7, 10.06)	1.49<br>(0.37, 3.41)	4.48<br>(3.08, 6.27)	11.11<br>(9.08, 13.3)	22.76<br>(18.13, 27.65)
Neurologic	Smell problems	1.5<br>(0.98, 2.16)	1.3<br>(0.79, 1.96)	3.24<br>(1.32, 6.83)	1.77<br>(0.85, 3.28)	1.38<br>(0.7, 2.37)	1.29<br>(0.43, 2.67)
Cardiovascular	Myocarditis	1<br>(0.61, 1.6)	1.02<br>(0.61, 1.66)	0.01<br>(0, 0.07)	0.53<br>(0.21, 1.27)	0.49<br>(0.21, 1.07)	1.12<br>(0.48, 2.44)
Dermatologic	Hair loss	0.43<br>(0.23, 0.7)	0.21<br>(0.05, 0.48)	8.48<br>(4, 15.12)	0.58<br>(0.18, 1.27)	0.56<br>(0.26, 1.01)	0<br>(-0.09, 0.35)

Post-acute sequelae were ascertained from 30 days after infection until end of follow-up.
Estimates of burdens per 1000 COVID-19 patients at 6-months are presented.

Supplementary Table 9: Burden of Post-acute Sequelae of COVID-19 individual sequelae in the overall cohort and by age, race, sex, and health status in COVID-19 admitted to intensive care

Organ	Sequelae	Overall	Age			Race	
			Age ≤ 60	Age >60 - ≤ 70	Age >70	Black	White
Pulmonary	Shortness of breath	99.81 (91.64, 108.18)	81.13 (67.03, 96.99)	98.42 (82.56, 115.75)	110.56 (98.49, 123.17)	96.67 (83.27, 111.23)	100.27 (89.91, 111.13)
Mental health	Sleep disorder	77.55 (67.33, 88.16)	109.41 (82.65, 138.81)	63.42 (44.52, 84.69)	76.07 (62.86, 90.28)	58.12 (42.47, 75.5)	91.81 (77.97, 106.39)
Endocrine	Hyperlipidemia	41.8 (31.14, 52.79)	55.66 (29.85, 83.98)	44.59 (22.66, 68.72)	36.1 (22.75, 50.5)	30 (14.13, 47.59)	48.58 (34.23, 63.65)
Cardiovascular	Chest pain	31.99 (26.64, 37.73)	47.99 (35.58, 62.53)	32.3 (21.72, 44.74)	24.39 (17.92, 31.71)	38.35 (28.47, 49.6)	29.45 (22.94, 36.67)
General	Fatigue	83.47 (76.6, 90.56)	44.41 (34.81, 55.59)	84.61 (71.43, 99.11)	109.34 (98.07, 121.23)	82.57 (71.24, 94.92)	81.42 (72.71, 90.58)
Gastrointestinal	GERD	67.61 (58.65, 76.96)	31.82 (18.35, 47.74)	66.46 (49.81, 85.16)	86.64 (72.85, 101.39)	56.84 (43.36, 71.94)	72.65 (60.65, 85.39)
Coagulation	Thromboembolism	64.14 (58.19, 70.5)	48.68 (39.08, 60.2)	66.47 (54.97, 79.73)	71.28 (62.37, 81.08)	60.83 (51.4, 71.59)	66.34 (58.46, 74.89)
Pulmonary	Cough	22.71 (18.47, 27.42)	31.7 (22.13, 43.66)	19.96 (12.42, 29.55)	19.9 (14.47, 26.28)	32.24 (24.12, 41.85)	16.1 (11.37, 21.63)
Musculoskeletal	Muscle weakness	60.24 (54.79, 65.96)	27.07 (20.58, 34.96)	55.09 (44.93, 66.63)	90.08 (80.18, 100.62)	57.19 (48.13, 67.32)	61.5 (54.46, 68.99)
Musculoskeletal	Joint pain	55.47 (47.24, 64.07)	41.05 (26.37, 58.04)	43.99 (29.19, 60.78)	69.43 (57.13, 82.64)	53.04 (39.21, 68.41)	57.03 (46.5, 68.26)
Mental health	Anxiety	76.72 (69.6, 84.11)	60.04 (46.04, 76.3)	83.38 (68.84, 99.52)	81.06 (71.47, 91.34)	73.86 (61.78, 87.18)	76.61 (67.69, 86.1)
Endocrine	Diabetes mellitus	51.03 (42.71, 60.02)	45.04 (32.16, 60.65)	48.61 (32.51, 68.14)	55.58 (42.95, 69.87)	57.54 (42.17, 75.49)	45.21 (35.44, 56.17)
Cardiovascular	Arrythmias	31.59 (26.69, 36.91)	27.47 (18.84, 38.28)	33.88 (24.65, 44.91)	31.74 (24.82, 39.61)	26.1 (18.68, 34.85)	33.23 (26.83, 40.38)
Mental health	Depression	37.56 (31.87, 43.66)	31.85 (19.24, 47.04)	41.95 (30.82, 54.91)	38.83 (31.3, 47.23)	27.1 (18.22, 37.5)	43.14 (35.61, 51.37)
Kidney	Chronic kidney disease	82.55 (71.93, 93.78)	13.37 (8.31, 19.86)	66.37 (49.47, 86.02)	169.58 (143.83, 196.91)	71.35 (57.93, 86.27)	83.77 (68.5, 100.4)
Gastrointestinal	Constipation	42.46 (37.21, 48.02)	27.59 (19.8, 37.1)	33.18 (24.15, 43.79)	56.92 (48.27, 66.22)	32.56 (24.27, 42.01)	49.64 (42.55, 57.29)
Neurologic	Memory problems	37.89 (33.13, 42.96)	17.68 (11.89, 25.27)	33.05 (24.77, 42.96)	63.39 (53.74, 73.86)	32.41 (25.62, 40.26)	40.99 (34.55, 48.06)
Pulmonary	Hypoxemia	37.73	23.39	41.22	48	29.48	42.24

		(33.88, 41.91)	(17.73, 30.54)	(33.58, 50.29)	(41.57, 55.13)	(23.76, 36.46)	(37.01, 48.01)
Cardiovascular	Acute coronary disease	50.33 (44.21, 56.8)	14.64 (9.59, 21.16)	51.24 (39.49, 64.74)	92.07 (78.3, 106.86)	43.69 (34.84, 53.69)	53.72 (45.23, 62.88)
Kidney	Acute kidney injury	73.18 (67.53, 79.02)	31.32 (25.16, 38.54)	75.08 (63.98, 87.38)	100.86 (91.03, 111.23)	92.16 (80.93, 104.43)	68.09 (61.16, 75.4)
Cardiovascular	Tachycardia	32.08 (28.01, 36.5)	34.76 (26.45, 44.87)	38.41 (30.23, 48.09)	26.55 (21.46, 32.39)	39.05 (31.51, 47.89)	28.69 (23.8, 34.19)
Gastrointestinal	Diarrhea	23.73 (19.86, 27.96)	17.37 (10.6, 26.13)	24.63 (17.55, 33.21)	25.72 (20.23, 31.97)	18.85 (13.29, 25.57)	25.9 (20.76, 31.7)
Mental health	Mood disorder	19.05 (14.73, 23.79)	19.73 (8.36, 33.93)	19.38 (11.61, 29.1)	18.89 (13.47, 25.22)	17.72 (9.91, 27.18)	19.35 (14.05, 25.39)
Neurologic	Headache	10.95 (7.87, 14.42)	20.63 (10.59, 33.19)	13.58 (7.09, 21.95)	8.49 (4.89, 13.01)	12.06 (6.39, 19.11)	10.92 (7.12, 15.44)
Endocrine	Obesity	19.59 (16.4, 22.98)	31.34 (21.44, 42.84)	20.71 (14.02, 28.54)	17.33 (13.55, 21.58)	15.91 (10.68, 21.99)	21.48 (17.34, 25.99)
Dermatologic	Skin rash	8.98 (5.75, 12.66)	11.3 (4.87, 20.06)	7.27 (1.71, 14.74)	8.8 (4.43, 14.14)	4.38 (0.12, 10.15)	10.97 (6.61, 16.1)
Mental health	Substance abuse	25.19 (20.16, 30.62)	30.92 (18.51, 45.95)	32.01 (21.11, 44.78)	20.33 (14.49, 27.05)	29.84 (20.54, 40.66)	22.03 (16.01, 28.79)
Neurologic	Stroke	16.78 (13.69, 20.23)	6.1 (3.42, 9.98)	16.85 (10.99, 24.4)	24.33 (18.67, 30.86)	24.31 (18.29, 31.48)	13.81 (10.19, 18.1)
Cardiovascular	Bradycardia	16.46 (13.35, 19.94)	8.27 (4.39, 14.17)	12.94 (7.82, 19.81)	25.19 (19.56, 31.67)	11.86 (7.65, 17.23)	19.3 (15, 24.29)
Cardiovascular	Heart failure	16.83 (14.45, 19.43)	6.15 (4.12, 8.92)	15.09 (10.72, 20.52)	27.52 (22.71, 32.9)	16.23 (12.28, 20.96)	17.18 (14.13, 20.61)
Neurologic	Smell problems	3.13 (1.95, 4.83)	3.57 (1.3, 8.33)	4 (1.82, 8.07)	2.25 (0.99, 4.51)	1.92 (0.54, 4.83)	3.45 (1.94, 5.83)
Cardiovascular	Myocarditis	2.13 (1.22, 3.66)	1.76 (0.62, 4.74)	2.32 (0.92, 5.69)	0.96 (0.35, 2.54)	1.62 (0.6, 4.19)	1.86 (0.88, 3.85)
Dermatologic	Hair loss	0.44 (0.11, 1.05)	1.14 (-0.08, 4.39)	0.61 (0.01, 2.49)	0.1 (0, 0.49)	0.48 (-0.05, 1.89)	0.4 (0.04, 1.37)

Organ	Sequelae	Overall	Sex		Comorbidity		
			Male	Female	No comorbidities	1-3 comorbidities	>3 comorbidities
Pulmonary	Shortness of breath	99.81 (91.64, 108.18)	100.2 (91.75, 108.88)	88.3 (61.05, 122.7)	90.99 (76.22, 107.56)	112.35 (99.5, 125.95)	129.5 (109.95, 150.31)
Mental health	Sleep disorder	77.55 (67.33, 88.16)	78.39 (67.87, 89.34)	65.1 (27.8, 113.78)	99.67 (78.58, 122.94)	66.93 (52.96, 82.06)	82.73 (62.29, 105.39)

Endocrine	Hyperlipidemia	41.8 (31.14, 52.79)	42.37 (31.43, 53.69)	29.43 (-9.62, 80.99)	69.21 (46.64, 93.88)	43.71 (27.83, 60.72)	20.97 (3.96, 40.03)
Cardiovascular	Chest pain	31.99 (26.64, 37.73)	31.89 (26.4, 37.79)	33.42 (13.39, 62.42)	29.47 (20.05, 41.03)	41.14 (32.7, 50.55)	25.11 (14.57, 37.44)
General	Fatigue	83.47 (76.6, 90.56)	86.42 (79.22, 93.88)	42.2 (25.07, 65.71)	61.61 (49.96, 75.1)	105.01 (93.27, 117.44)	129.56 (111.33, 149.06)
Gastrointestinal	GERD	67.61 (58.65, 76.96)	68.59 (59.32, 78.28)	55.93 (25.2, 97.03)	37.16 (23.48, 53.23)	71.6 (58.39, 85.9)	90.2 (71.33, 110.89)
Coagulation	Thromboembolism	64.14 (58.19, 70.5)	67.41 (61.1, 74.15)	20.81 (10.03, 38.99)	67.07 (54.6, 81.62)	78.55 (68.44, 89.7)	71.03 (58.97, 84.7)
Pulmonary	Cough	22.71 (18.47, 27.42)	21.59 (17.32, 26.37)	42.89 (22.7, 72.91)	23.03 (15.2, 33.27)	24.13 (17.81, 31.58)	23.3 (14.91, 33.45)
Musculoskeletal	Muscle weakness	60.24 (54.79, 65.96)	63.1 (57.32, 69.17)	24.99 (13.59, 41.74)	45.53 (36.18, 56.55)	77.24 (67.49, 87.77)	105.87 (90.07, 123.02)
Musculoskeletal	Joint pain	55.47 (47.24, 64.07)	57.12 (48.58, 66.05)	31.51 (5.71, 67.73)	43.16 (29.69, 58.71)	56.66 (44.76, 69.61)	73.57 (54.6, 94.51)
Mental health	Anxiety	76.72 (69.6, 84.11)	77.46 (70.12, 85.09)	63.96 (37.21, 100.35)	68.05 (54.62, 83.52)	80.44 (69.74, 92)	98.12 (82.99, 114.62)
Endocrine	Diabetes mellitus	51.03 (42.71, 60.02)	54.14 (45.25, 63.75)	17.52 (2.28, 44.83)	61.8 (48.88, 76.33)	41.16 (29.46, 54.86)	45.21 (24.57, 72.31)
Cardiovascular	Arrythmias	31.59 (26.69, 36.91)	31.81 (26.77, 37.32)	24.93 (8.9, 50.51)	27.9 (19.26, 38.86)	35.57 (27.99, 44.22)	34.87 (24.8, 46.58)
Mental health	Depression	37.56 (31.87, 43.66)	36.76 (31.02, 42.92)	50.22 (22.54, 89.04)	32.43 (22.03, 45.09)	42.11 (33.6, 51.62)	40.73 (29.39, 53.69)
Kidney	Chronic kidney disease	82.55 (71.93, 93.78)	86.89 (75.57, 98.86)	33.75 (12.87, 67.62)	49.25 (36.08, 65.16)	107.91 (89.86, 127.46)	131.26 (98.06, 168.59)
Gastrointestinal	Constipation	42.46 (37.21, 48.02)	43.43 (37.99, 49.19)	28.77 (11.43, 54.27)	30.78 (22.33, 41.17)	56.66 (47.77, 66.42)	51.13 (38.58, 65.13)
Neurologic	Memory problems	37.89 (33.13, 42.96)	39.04 (34.01, 44.41)	26.21 (14.17, 44.5)	35.68 (27.25, 45.89)	45.36 (37.48, 54.2)	46.41 (35.39, 58.93)
Pulmonary	Hypoxemia	37.73 (33.88, 41.91)	39.66 (35.55, 44.15)	14.25 (7.96, 24.49)	36.03 (28.25, 45.7)	51.24 (44.09, 59.31)	54.54 (44.67, 65.93)
Cardiovascular	Acute coronary disease	50.33 (44.21, 56.8)	56.32 (49.48, 63.57)	4.79 (-0.24, 14.73)	37.36 (28.68, 47.63)	64.27 (53.65, 75.88)	71.93 (54.64, 91.33)
Kidney	Acute kidney injury	73.18 (67.53, 79.02)	78.82 (72.69, 85.16)	14.09 (7.5, 24.18)	52.18 (42.27, 63.75)	102.76 (92.06, 114.18)	153.68 (134.48, 174.16)
Cardiovasc	Tachycardia	32.08	32.05	31.06	40.9	39.7	29.8

ular		(28.01, 36.5)	(27.86, 36.6)	(17, 52.54)	(31.52, 52.34)	(32.86, 47.48)	(22.1, 39)
Gastrointestinal	Diarrhea	23.73<br>(19.86, 27.96)	24.17<br>(20.15, 28.58)	18.25<br>(6.22, 37.33)	16.01<br>(9.96, 24.22)	28.17<br>(21.98, 35.35)	33.4<br>(24.11, 44.15)
Mental health	Mood disorder	19.05<br>(14.73, 23.79)	18.52<br>(14.15, 23.35)	30.42<br>(10.07, 60.75)	16.13<br>(8.1, 26.45)	16.04<br>(10.26, 22.89)	25.78<br>(17.4, 35.75)
Neurologic	Headache	10.95<br>(7.87, 14.42)	10.8<br>(7.68, 14.36)	21.69<br>(1.95, 51.93)	16.31<br>(9.47, 25.16)	10.88<br>(6.59, 16.14)	7.41<br>(2.42, 13.93)
Endocrine	Obesity	19.59<br>(16.4, 22.98)	19.51<br>(16.31, 22.94)	19.24<br>(3.6, 42.16)	24.02<br>(17.48, 31.71)	20.04<br>(15.31, 25.33)	19.32<br>(13, 26.68)
Dermatologic	Skin rash	8.98<br>(5.75, 12.66)	8.06<br>(4.83, 11.78)	23.73<br>(8.36, 47.95)	7.84<br>(2.4, 15.72)	11.37<br>(6.47, 17.4)	7.28<br>(1.28, 14.95)
Mental health	Substance abuse	25.19<br>(20.16, 30.62)	25.63<br>(20.41, 31.28)	17.72<br>(3.23, 41.7)	29.63<br>(18.87, 42.77)	24.01<br>(16.99, 32.04)	25.39<br>(16.48, 35.92)
Neurologic	Stroke	16.78<br>(13.69, 20.23)	18.15<br>(14.8, 21.88)	1.95<br>(-0.93, 9.65)	16.34<br>(10.94, 23.67)	18.02<br>(13.12, 23.96)	25.59<br>(17.5, 35.28)
Cardiovascular	Bradycardia	16.46<br>(13.35, 19.94)	17.38<br>(14.06, 21.1)	5.06<br>(0.37, 16.39)	16.37<br>(10.61, 24.28)	20.38<br>(15.32, 26.47)	17.81<br>(11.12, 26.05)
Cardiovascular	Heart failure	16.83<br>(14.45, 19.43)	18.37<br>(15.75, 21.23)	2.42<br>(0.48, 6.79)	11.38<br>(7.51, 16.81)	21.54<br>(17.02, 26.81)	47.64<br>(37.77, 58.81)
Neurologic	Smell problems	3.13<br>(1.95, 4.83)	3.21<br>(1.97, 5.01)	2.21<br>(0.1, 10.84)	3.76<br>(1.53, 8.37)	2.98<br>(1.43, 5.69)	2.84<br>(1.04, 6.3)
Cardiovascular	Myocarditis	2.13<br>(1.22, 3.66)	2.1<br>(1.18, 3.68)	0.03<br>(0, 0.28)	1.05<br>(0.31, 3.51)	1.6<br>(0.7, 3.47)	1.81<br>(0.65, 4.61)
Dermatologic	Hair loss	0.44<br>(0.11, 1.05)	0.16<br>(-0.04, 0.82)	9.82<br>(2.35, 26.26)	0.98<br>(0.17, 3.13)	0.39<br>(0.01, 1.41)	0.06<br>(-0.1, 1.21)
Post-acute sequelae were ascertained from 30 days after infection until end of follow-up.<br>Estimates of burdens per 1000 COVID-19 patients at 6-months are presented.							

Supplementary Table 10: Overall burden of Post-Acute Sequelae of SARS-CoV-2 infection (PASC) after 12 weeks per 1000 persons at 6 months in the overall cohort and across care setting (non-hospitalized, hospitalized, and admitted to intensive care during the acute phase of the infection).

	COVID-19 group Adjusted burden per 1000 persons at 6 months (95% CI)	Users of the VHA without COVID-19 Adjusted burden per 1000 persons at 6 months (95% CI)	Burden associated with COVID-19† Adjusted burden per 1000 persons at 6 months (95% CI)
Overall	145.76 (144.07, 147.36)	86.36 (85.97, 86.77)	59.36 (57.72, 60.97)
Non-hospitalized COVID-19*	126.96 (125.17, 128.67)	86.36 (85.97, 86.77)	40.60 (38.83, 42.26)
Hospitalized COVID-19*	244.63 (239.22, 250.68)	86.36 (85.97, 86.77)	158.31 (152.92, 164.30)
COVID-19 required ICU*	313.58 (301.92, 324.91)	86.36 (85.97, 86.77)	227.18 (215.65, 238.46)

*. Care settings during the first 30 days of infection.
†. Burden defined as having at least one sequela in excess of users of the Veterans Health Administration without COVID-19 after the first 84-days of infection. Burden was estimated based on Poisson regression.

Supplementary Table 11: Risks and burdens of individual sequela after 12 weeks infection of COVID-19

Organ	Sequelae	Hazard Ratio (95% CI)	Burden per 1000 persons at 6 months (95% CI)
Cardiovascular	Chest pain	1.43 (1.39, 1.46)	12.61 (11.53, 13.62)
	Arrythmias	1.34 (1.29, 1.38)	6.33 (5.49, 7.12)
	Acute coronary disease	1.17 (1.13, 1.21)	4.78 (3.84, 5.66)
	Tachycardia	1.26 (1.19, 1.33)	2.17 (1.67, 2.65)
	Bradycardia	1.2 (1.14, 1.27)	1.94 (1.37, 2.48)
	Heart failure	1.14 (1.07, 1.21)	1.13 (0.74, 1.52)
	Myocarditis	4.75 (3.22, 6.8)	0.22 (0.14, 0.32)
Coagulation	Thromboembolism	1.68 (1.6, 1.76)	7.44 (6.71, 8.14)
Dermatologic	Skin rash	1.18 (1.14, 1.22)	3.09 (2.38, 3.78)
	Hair loss	1.83 (1.63, 2.05)	0.27 (0.2, 0.34)
Endocrine	Hyperlipidemia	1.07 (1.06, 1.09)	14.74 (11.35, 17.85)
	Diabetes mellitus	1.21 (1.17, 1.24)	6.98 (5.82, 8.07)
	Obesity	1.16 (1.13, 1.19)	3.01 (2.52, 3.48)
Gastrointestinal	GERD	1.18 (1.16, 1.21)	10.44 (9.01, 11.77)
	Constipation	1.22 (1.18, 1.26)	5.16 (4.24, 6.01)
	Diarrhea	1.28 (1.23, 1.33)	4.13 (3.44, 4.79)
General	Fatigue	1.29 (1.25, 1.33)	7.84 (6.86, 8.77)
Kidney	Chronic kidney disease	1.15 (1.11, 1.20)	5.76 (2.68, 8.68)
	Acute kidney injury	1.16 (1.11, 1.21)	3.02 (2.34, 3.67)
Mental health	Sleep disorders	1.25 (1.23, 1.28)	17.86 (16.22, 19.39)
	Anxiety	1.12 (1.08, 1.15)	5.24 (3.81, 6.57)
	Depression	1.19 (1.15, 1.22)	5.58 (4.66, 6.46)
	Mood disorder	1.17 (1.14, 1.21)	3.79 (3.05, 4.49)
	Substance abuse	1.11 (1.06, 1.15)	1.27 (0.8, 1.82)
Musculoskeletal	Muscle weakness	1.3 (1.25, 1.35)	5.32 (4.53, 6.06)
	Joint pain	1.11 (1.08, 1.13)	6.61 (5.18, 7.94)
Neurologic	Memory problems	1.35 (1.3, 1.4)	5.92 (5.13, 6.67)
	Headache	1.27 (1.23, 1.31)	3.88 (3.31, 4.42)
	Stroke	1.26 (1.19, 1.32)	2.36 (1.8, 2.9)
	Smell problems	3.45 (3.11, 3.8)	1.74 (1.5, 1.98)
Pulmonary	Shortness of breath	1.72 (1.68, 1.76)	24.13 (22.83, 25.34)

	Cough	1.47 (1.42, 1.52)	7.5 (6.68, 8.27)
	Hypoxemia	1.77 (1.67, 1.88)	4.08 (3.62, 4.53)
Adjusted hazard ratio, adjusted burden per 1000 persons at 6 months of people with COVID-19 compared to users of the Veterans Health Administration. All users of the VHA served as the referent category. Outcomes were ascertained from week 12 after COVID-19 diagnosis until end of follow-up. For each outcome, cohort participants without history of the outcome in past one year were included in the analysis.			

Supplementary Table 12: Results of negative outcome controls in people with COVID-19 (the overall COVID-19 cohort), and in non-hospitalized, hospitalized, and admitted to intensive care for COVID-19.

Negative outcome control	COVID-19 Hazard Ratio (95% CI)	Care setting*		
		Non-hospitalized COVID-19 Hazard Ratio (95% CI)	Hospitalized COVID-19 Hazard Ratio (95% CI)	COVID-19 required ICU Hazard Ratio (95% CI)
Accidental injuries †	1.04 (0.84, 1.29)	1.03 (0.88, 1.22)	1.06 (0.68, 1.63)	1.07 (0.56, 2.03)
Neoplasms †	1.01 (0.88, 1.17)	1.04 (0.94, 1.06)	0.87 (0.79, 1.16)	0.88 (0.76, 1.25)

∗. Care setting during the first 30 days of infection.
†. The users of the Veterans Health Administration without COVID-19 served as the referent category. Outcomes ascertained after the first 30-days of infection.

Supplementary Table 13: Definition of each Post-Acute Sequela of SARS-CoV-2 infection (PASC)

Organ	Sequelae	Definition
Cardiovascular	Acute coronary disease	ICD10 code: I21, I22, I24, I2510, I2511
	Arrythmias	ICD10 code: I49, R002, R008, R009
	Bradycardia	ICD10 code: R001
	Chest pain	ICD10 code: R071, R078, R079
	Heart failure	ICD10 code: I5021, I5023, I5031, I5033, I5041, I5043, I50811, I50813
	Myocarditis	ICD10 code: B3320, B3321, B3322, B3323, B3324, I400
	Tachycardia	ICD10 code: R000
Coagulation	Thromboembolism	ICD10 code: I740-I745, I748, I749, I800-I803, I808-I819, I820-I829, I82A-I82C, I2601, I2602, I2609, I2690, I2692, I2693, I2694, I2699
Dermatologic	Hair loss	ICD10 code: L659, L658, L63
	Skin rash	ICD10 code: R21, B09, L282
Endocrine	Diabetes mellitus	ICD10 code: E08, E09, E10, E11, E13 or HbA1c > 6.5% or use of antihyperglycemics
	Hyperlipidemia	Low-density lipoprotein cholesterol>130 mg/dL or Triglycerides>150 mg/dL and use of antilipemic agents
	Obesity	ICD10 code: E668, E669, E661, E662 or BMI>30 kg/m^2
Gastrointestinal	Constipation	ICD10 code: K5900, K5901, K5902, K5903, K5904, K5909 or use of laxatives
	Diarrhea	ICD10 code: R197
	GERD	ICD10 code: K210, K2100, K2101, K219
General	Fatigue	ICD10 code: R531, R5381, R5382, R8383
Kidney	Acute kidney injury	Serum creatine measurement increases more than 50% or increases 0.3 mg/dL
	Chronic kidney disease	eGFR measurement < 60 $mL/min/1.73m^2$
Mental health	Anxiety	Use of benzodiazepine derivative sedatives or hypnotics
	Depression	ICD10 code: F320, F321, F322, F323, F324, F329
	Mood disorder	ICD10 code: F23, F24, F28, F4310, F0631, F0632, F063, F341
	Sleep disorder	ICD10 code: F510, F511, F513-F515, F518, F519, G470-G476. G478, G479, R063
	Substance abuse	ICD10 code: F10-F19
Musculoskeletal	Joint pain	ICD10 code: M25.5, M79.6, R52, G89, M00-M14
	Muscle weakness	ICD10 code: M6281
Neurologic	Headache	ICD10 code: G439, G43C, G440, G442, R51
	Memory problems	ICD10 code: R411-R413, F0150, F0151, F0280, F0281, F0390, F0391, F04, F05, F060, F068, G309, G3109, G311, G3183-G3185, G3189, G319, G910, G911, G937, G94
	Smell problems	ICD10 code: R430, R431, R432, R438, R439
	Stroke	ICD10 code: I514, I60-I62, I630-I636, I638, I639

Pulmonary	Cough	ICD10 code: R05 or use of antitussives or expectorants
	Hypoxemia	ICD10 code: R0902
	Shortness of breath	ICD10 code: R0600, R0602, R0609
Each PASC was defined as occurrence of outcome between 30 days after infection and the end of follow up, within those without the occurrence of outcome within one year before infection.		
