# Supplementary material for: Burdens of post-acute sequelae of COVID-19 by severity of acute infection, demographics and health status
Source: Nat Commun. 2021 Nov 12;12:6571. doi: 10.1038/s41467-021-26513-3 (PMC8589966; doi:10.1038/s41467-021-26513-3)
Supplement: Supplementary file 2 — Reporting summary. [file 41467_2021_26513_MOESM2_ESM.pdf]

Corresponding author(s): Ziyad Al-Aly

Last updated by author(s): Oct 10, 2021

Reporting Summary

Nature Research wishes to improve the reproducibility of the work that we publish. This form provides structure for consistency and transparency in reporting. For further information on Nature Research policies, see our Editorial Policies and the Editorial Policy Checklist.

Statistics

For all statistical analyses, confirm that the following items are present in the figure legend, table legend, main text, or Methods section.

n/a	Confirmed	
☐	☒	The exact sample size (n) for each experimental group/condition, given as a discrete number and unit of measurement
☐	☒	A statement on whether measurements were taken from distinct samples or whether the same sample was measured repeatedly
☒	☐	The statistical test(s) used AND whether they are one- or two-sided<br>*Only common tests should be described solely by name; describe more complex techniques in the Methods section.*
☐	☒	A description of all covariates tested
☐	☒	A description of any assumptions or corrections, such as tests of normality and adjustment for multiple comparisons
☐	☒	A full description of the statistical parameters including central tendency (e.g. means) or other basic estimates (e.g. regression coefficient) AND variation (e.g. standard deviation) or associated estimates of uncertainty (e.g. confidence intervals)
☒	☐	For null hypothesis testing, the test statistic (e.g. F, t, r) with confidence intervals, effect sizes, degrees of freedom and P value noted<br>*Give P values as exact values whenever suitable.*
☒	☐	For Bayesian analysis, information on the choice of priors and Markov chain Monte Carlo settings
☒	☐	For hierarchical and complex designs, identification of the appropriate level for tests and full reporting of outcomes
☒	☐	Estimates of effect sizes (e.g. Cohen's d, Pearson's r), indicating how they were calculated

Our web collection on statistics for biologists contains articles on many of the points above.

Software and code

Policy information about availability of computer code

Data collection	SAS Enterprise Guide version 7.1 was used to collect data for the study.
Data analysis	All analyses were done using SAS Enterprise Guide version 7.1 (SAS Institute, Cary, NC). Data visualizations were performed in R 4.0.3 (R Foundation for Statistical Computing, Vienna, Austria). Code used for this study can be found at https://github.com/yxie618/longPASC

For manuscripts utilizing custom algorithms or software that are central to the research but not yet described in published literature, software must be made available to editors and reviewers. We strongly encourage code deposition in a community repository (e.g. GitHub). See the Nature Research guidelines for submitting code & software for further information.

Data

Policy information about availability of data

All manuscripts must include a data availability statement. This statement should provide the following information, where applicable:

- Accession codes, unique identifiers, or web links for publicly available datasets
- A list of figures that have associated raw data
- A description of any restrictions on data availability

The data that support the findings of this study are available from the VA. VA data are made freely available to researchers behind the VA firewall with an approved VA study protocol. More information is available at https://www.virec.research.va.gov or by contacting the VA Information Resource Center (VIReC) at VIReC@va.gov.

Field-specific reporting

Please select the one below that is the best fit for your research. If you are not sure, read the appropriate sections before making your selection.

- [x] Life sciences
- [ ] Behavioural & social sciences
- [ ] Ecological, evolutionary & environmental sciences

For a reference copy of the document with all sections, see nature.com/documents/nr-reporting-summary-flat.pdf

Life sciences study design

All studies must disclose on these points even when the disclosure is negative.

Sample size	To achieve better precision, we enrolled all users of the US Veterans Health Administration and followed them until March 15, 2021. This cohort included 155,987 people who were not hospitalized during the acute phase of COVID-19, 19,359 people who were hospitalized during the acute phase of COVID-19, 6038 people who were admitted to intensive care during the acute phase of COVID-19 and 4,397,509 non-infected controls.
Data exclusions	To examine the risk of post-acute outcomes beyond the first 30 days of illness, we predefined our exclusion criteria and excluded participants who did not survive the first 30 days of COVID-19 illness.
Replication	The findings were consistent in additional analyses which defined post-acute sequelae as incident clinical manifestations in excess of the non-infected controls that occurred after 12 weeks (84 days) from the COVID-19 positive test.
Randomization	We conducted an observational study. Exposure allocation was not random.
Blinding	We conducted an observational study. Blinding was not possible.

Reporting for specific materials, systems and methods

We require information from authors about some types of materials, experimental systems and methods used in many studies. Here, indicate whether each material, system or method listed is relevant to your study. If you are not sure if a list item applies to your research, read the appropriate section before selecting a response.

Materials & experimental systems

n/a	Involved in the study	
x		Antibodies
x		Eukaryotic cell lines
x		Palaeontology and archaeology
x		Animals and other organisms
	x	Human research participants
x		Clinical data
x		Dual use research of concern

Methods

n/a	Involved in the study	
x		ChIP-seq
x		Flow cytometry
x		MRI-based neuroimaging

Human research participants

Policy information about studies involving human research participants

Population characteristics	Study participants are users of the US Veteran Health Administration. The overall study population had a median age of 67, 90% were male, and 76% of cohort participants were of white race; 155,987, 19,359 and 6038 were non-hospitalized, hospitalized, and admitted to intensive care during the acute phase of COVID-19, respectively; there were 4,397,509 non-infected controls.
Recruitment	Participants were recruited if they had at least 1 encounter with the US Veteran Health Administration in year 2019. Non users of the VA health care system were not included. The characteristics of the study population may be different from the general population (US or global population). Other biases due to recruitment including self-selection are unlikely to bias the results of this study.
Ethics oversight	The study was approved by the Institutional Review Board of the Veterans Affairs St. Louis Health Care System, St. Louis, MO, US

Note that full information on the approval of the study protocol must also be provided in the manuscript.
